# DADA Enhances CD8^+^ T Cell Stemness to Improve Anti‐Tumor Immunity and Immunotherapy Efficacy

**DOI:** 10.1002/advs.202519765

**Published:** 2026-02-27

**Authors:** Mingyue Bi, Fei Li, Heng Jiang, Lingling Gu, Mengjie Sun, Kunyu Lu, Pianpian Liu, Ruyue Xie, Wenqiang Ma, Meijuan Zheng, Xianwei Wang, Xuefu Wang, Jingjing Cong

**Affiliations:** ^1^ Department of Clinical Laboratory First Affiliated Hospital of Anhui Medical University Hefei Anhui China; ^2^ School of Pharmacy, Inflammation and Immune Mediated Diseases Laboratory of Anhui Province Anhui Medical University Hefei Anhui China; ^3^ Department of General Surgery The Second Affiliated Hospital of Anhui Medical University Hefei China; ^4^ Department of Clinical Laboratory The Second Affiliated Hospital of Anhui Medical University Hefei China; ^5^ State Key Laboratory of Immune Response and Immunotherapy, the Institute of Immunology, School of Basic Medical Sciences, Division of Life Sciences and Medicine University of Science and Technology of China Hefei Anhui China

**Keywords:** CD8^+^ T cell, anti‐tumor immunity, metabolic reprogramming, stemness

## Abstract

Progenitor exhausted CD8^+^ T (Tpex) cells have recently been identified as a stem‐like T cell subset that mediates durable anti‐tumor immune responses and represents a pivotal population responsive to immunotherapies. Here, it is demonstrated that diisopropylamine dichloroacetate (DADA) facilitates CD8^+^ T cell‐mediated anti‐tumor immunity and promotes Tpex cells accumulation in the tumor microenvironment. Mechanistically, DADA promotes the conversion from pyruvate to Acetyl‐CoA by inhibiting pyruvate dehydrogenase kinase. This process leads to increased oxidative phosphorylation (OXPHOS) and mitochondrial fitness, thereby enhancing CD8^+^ T cells stemness. Treatment of mice with DADA improves the efficacy of PD‐1 blockade. Furthermore, the in vitro expansion of chimeric antigen receptor (CAR)‐T cells supplemented with DADA confers them with stemness characteristics, contributing to improved anti‐tumor efficacy. Collectively, this study illustrates how DADA‐mediated metabolic reprogramming in CD8^+^ T cell enhances their stemness, underscoring its potential for anti‐tumor therapy.

## Graphical abstract

Diisopropylamine dichloroacetate (DADA) enhances CD8^+^ T cell stemness by improving OXPHOS and mitochondrial fitness in a PDK1‐depenpendent manner. This metabolic shift strengthens CD8^+^ T cell anti‐tumor immunity, improves responses to PD‐1 blockade, and endows CAR‐T cells with prolonged persistence and resistance to terminal exhaustion, highlighting a metabolic strategy to potentiate immunotherapy efficacy.

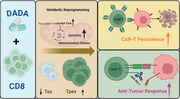

## 1 Introduction

CD8^+^ T cells are cytotoxic lymphocytes with a crucial role in resistance against various cancers through secretion of effector cytokines, including interferon (IFN)γ and tumor necrosis factor (TNF)‐α, as well as through direct killing of tumor cells. However, continuous exposure to persistent antigen stimulation drives the differentiation of CD8^+^ T cell toward exhaustion, manifesting hierarchical functional states [1, 2]. Within the tumor microenvironment, two distinct subsets of exhausted CD8^+^ T cells with different functional characteristics have been identified: terminally exhausted CD8^+^ T (Tex) cells and progenitor exhausted CD8^+^ T (Tpex) cells [3, 4, 5, 6].

Tex cells are characterized by high expression of multiple inhibitory receptors such as PD‐1, TIM‐3, LAG‐3, TIGIT and CD39, reduced production of effector cytokines, and impaired survival capacity [1]. Immune checkpoint blockade (ICB) and adoptive cell transfer (ACT) immunotherapies have emerged as significant breakthroughs in oncology in the last decades. However, the resistance of some patients to ICB and ACT therapies can be attributed to the terminal exhaustion of T cells, as reinvigorating Tex cells through ICB remains a huge challenge and the anti‐tumor efficacy of adoptively transferred T cells can be hindered by their transition into a terminally exhausted state [7, 8].

Tex cells stem from a stem‐like T cell population known as Tpex cells, typically defined by a phenotype of TCF1^+^TIM‐3^−^ [1, 2]. The stem‐like properties of Tpex cells are maintained by several transcription factors, such as TCF1, BACH2, and BCL6 [4, 9, 10, 11]. Tpex cells mediate more durable anti‐tumor immune responses compared to their TCF1^−^ effector‐like counterparts or Tex cells [3, 12]. Moreover, Tpex cells constitute a crucial population responsive to anti‐PD‐1/PD‐L1 therapy and ACT therapy [2, 3, 4, 13, 14, 15, 16]. Thus, preserving the stemness of CD8^+^ T cells or preventing the differentiation from Tpex cells into Tex cells could represent a promising approach to improve T cell anti‐tumor efficacy.

Cellular metabolism plays a crucial role in orchestrating fundamental biological processes that facilitate the adaptation of CD8^+^ T cells across different stages of activation and differentiation [17, 18]. Effector cells augment glycolysis and oxidative phosphorylation (OXPHOS) to support their rapid proliferation and cytotoxic capabilities [19]. Tex cells exhibit metabolic insufficiency, including impaired mitochondrial respiration and glycolysis, as well as increased mitochondrial ROS production [20, 21, 22, 23, 24], whereas Tpex cells sustain higher mitochondrial fitness and OXPHOS to ensure long‐term proliferative potential and multipotency [25]. However, the precise manipulation of cellular metabolism to regulate the stemness program of CD8^+^ T cells remains poorly understood.

Diisopropylamine dichloroacetate (DADA), the active component of pangamic acid [26], is an effective inhibitor of pyruvate dehydrogenase (PDH) kinase (PDK), which phosphorylates and inactivates pyruvate dehydrogenase complex (PDC) [27]. PDC functions in the mitochondrion to catalyze the irreversible decarboxylation of pyruvate to AcetylCoA, thereby shifting cellular metabolism from glycolysis toward the tricarboxylic acid cycle (TCA) and subsequent OXPHOS [28]. DADA has been utilized in clinical settings for numerous years as a hepatoprotective drug with a favorable safety profile by improving energy metabolism in hepatocytes [29], while its anti‐tumor properties against various types of tumor cells by inhibiting glycolysis have been acknowledged [30, 31]. Nevertheless, the potential impact of DADA on the cellular metabolism and stemness of CD8^+^ T cells, and how this might influence their anti‐tumor immune responses, remains largely unknown.

Here, we demonstrate that DADA boosts CD8^+^ T cell‐mediated anti‐tumor immunity and improves the effectiveness of immunotherapies. This occurs by enhancing CD8^+^ T cell stemness through promoting OXPHOS in a PDK1‐dependent manner. Our study uncovers a previously unknown mechanism for enhancing CD8^+^ T cell stemness and suggests the potential of utilizing DADA in anti‐tumor therapy.

## 2 Results

### 2.1 DADA Potentiates CD8^+^ T Cell Anti‐Tumor Immune Responses

**FIGURE 1:**
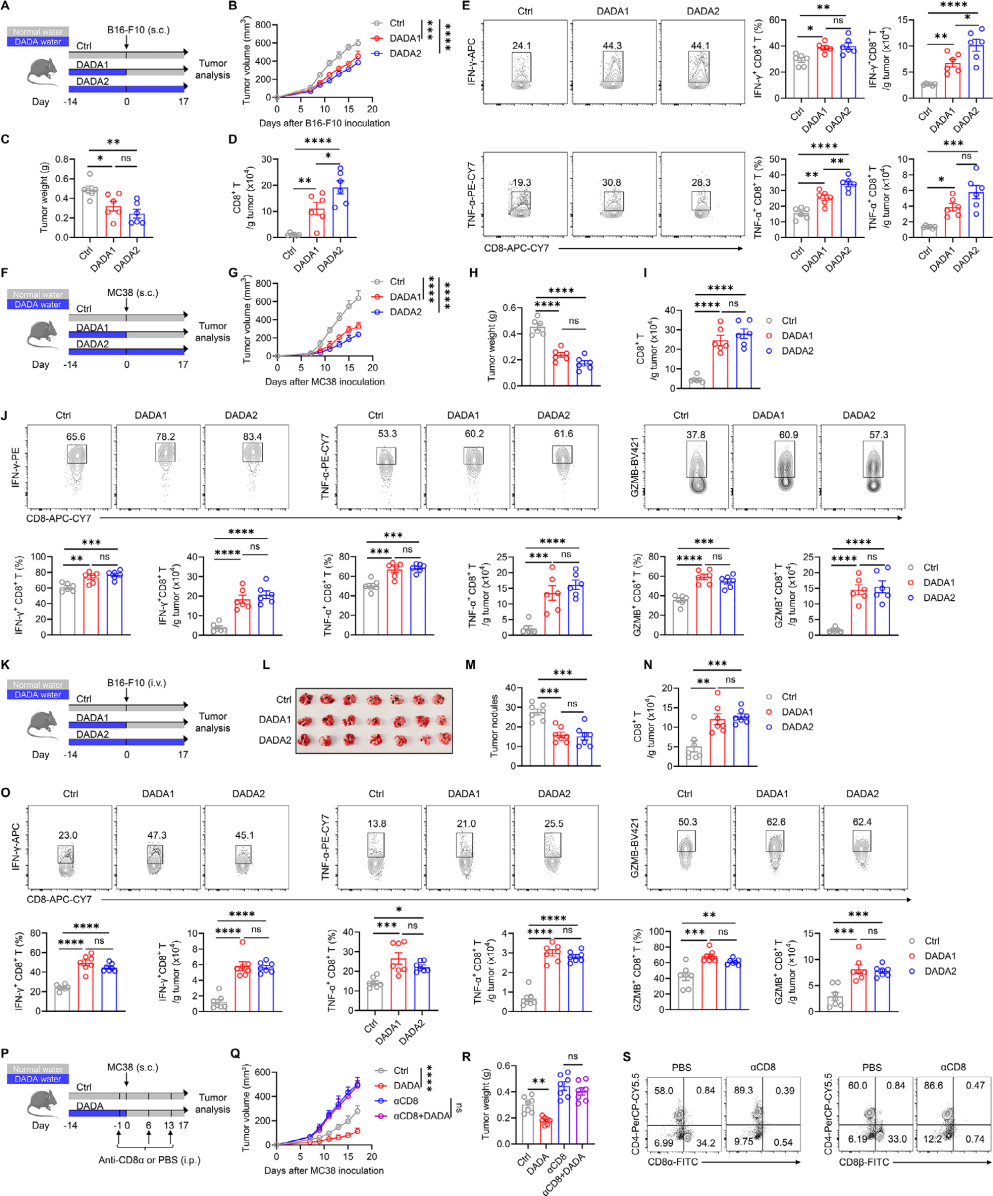
DADA inhibits tumor growth by potentiating CD8^+^ T cell anti‐tumor immune responses. (A) Schematic experimental procedure in (B–E): WT mice were fed with DADA‐containing or normal water from day ‐14 until the experimental endpoints or until day 0, and were injected (subcutaneously [s.c.]) with 16‐F10 cells on day 0. (B,C) Tumor growth curves (B) and tumor weights (C) at 17 days after B16‐F10 inoculation; *n* = 6. The experiment was repeated three times. (D,E) Representative flow cytometry plots and quantification of CD8^+^ T (D), IFN‐γ^+^CD8^+^ T and TNF‐α^+^CD8^+^ T (E) cells from tumor; *n* = 6. The experiment was repeated twice. (F) Schematic experimental procedure for (G–J): WT mice were fed with DADA‐containing or normal water from day ‐14 until the experimental endpoints or until day 0, and were injected (s.c.) with MC38 cells on day 0. (G and H) Tumor growth curves (G) and tumor weights (H) at 17 days after MC38 inoculation; *n* = 6. The experiment was repeated three times. (I,J) Representative flow cytometry plots and quantification of CD8^+^ T (I), IFN‐γ^+^CD8^+^ T, TNF‐α^+^CD8^+^ T and GZMB^+^CD8^+^ T (J) cells from tumor; n = 6. The experiment was repeated twice. (K) Schematic experimental procedure for (K–O): WT mice were fed with DADA‐containing or normal water from day ‐14 until the experimental endpoints or until day 0, and were injected (intravenously [i.v.]) with B16‐F10 cells on day 0. (L,M) Appearance of lungs (L) and the number of tumor nodules (M) at 17 days after B16‐F10 inoculation; *n* = 7. The experiment was repeated twice. (N and O) Representative flow cytometry plots and quantification of CD8^+^ T (N), IFN‐γ^+^CD8^+^ T, TNF‐α^+^CD8^+^ T and GZMB^+^CD8^+^ T (O) cells from the lung; *n* = 7. The experiment was repeated twice. (P) Schematic experimental procedure for (Q–S): WT mice were fed with DADA‐containing or normal water for 14 days and were injected (intraperitoneally [i.p.]) with 100 µg of anti‐CD8α antibody weekly. The mice were injected (s.c.) with MC38 cells on day 0. (Q–S) Tumor growth curves (Q), tumor weights (R), and representative flow cytometry plots of CD8^+^ T cells (S) at 17 days after MC38 inoculation; *n* = 7. The experiment was repeated three times. Data are presented as mean ± SD and are analyzed by two‐way ANOVA (B, G, and Q) and one‐way ANOVA (C–E, H–J, M–O, and R); *p < 0.05, **p < 0.01, ***p < 0.001, ****p < 0.0001. ns, not significant.

To investigate the effect of DADA on CD8^+^ T cell‐mediated anti‐tumor immune responses, we employed different subcutaneous tumor models, as well as a metastatic tumor model. Wild‐type (WT) mice were fed with DADA‐containing drinking or normal water for 2 weeks, and were subcutaneously injected with B16‐F10 melanoma tumor cells. The mice were then maintained on either DADA‐containing drinking or normal water for another 17 days (Figure 1A). We found that DADA exhibited safety profiles while notably inhibiting tumor growth (Figure 1B,C; Figure S1A,B). Notably, this anti‐tumor effect was not solely attributable to DADA's reported direct hindrance of tumor cell proliferation, as evidenced by the continued inhibition of tumor growth by DADA even when its administration ceased before the injection of B16‐F10 cells (Figure 1B,C), suggesting an intrinsic influence of DADA on host immunity.

Consistently, we observed increased cell counts of tumor‐infiltrating CD8^+^ T cells and higher expression of IFN‐γ and TNF‐α in these cells in DADA‐treated mice (Figure 1D,E; Figure S1C). Furthermore, there was an increase in the proportions of CD8^+^ T cells expressing IFN‐γ and TNF‐α in the tumor‐draining lymph nodes (TDLN) of DADA‐treated mice (Figure S1D). In both the MC38 subcutaneous tumor model and B16‐F10 lung metastasis model, either pretreatment or continuous treatment of DADA also led to inhibited tumor growth (Figure 1F–H, and K–M). This was accompanied by increased cell counts and enhanced effector functions of CD8^+^ T cells harvested from the tumor microenvironment and TDLN (Figure 1I,J,N,O; Figure S1E,F).

To confirm the dependency of DADA's anti‐tumor effect on CD8^+^ T cells, we injected the WT mice intraperitoneally with anti‐CD8α‐depleting antibody weekly to deplete CD8^+^ T cells (Figure 1P). In the absence of CD8^+^ T cells, DADA treatment did not inhibit tumor growth (Figure 1Q–S). However, the depletion of either CD4^+^ T cells or NK cells did not abolish the anti‐tumor effect of DADA (Figure S1G–K). These results collectively suggest that DADA inhibits tumor growth by enhancing CD8^+^ T cell anti‐tumor responses.

### 2.2 DADA Prevents Terminal Exhaustion of CD8^+^ T Cells

**FIGURE 2:**
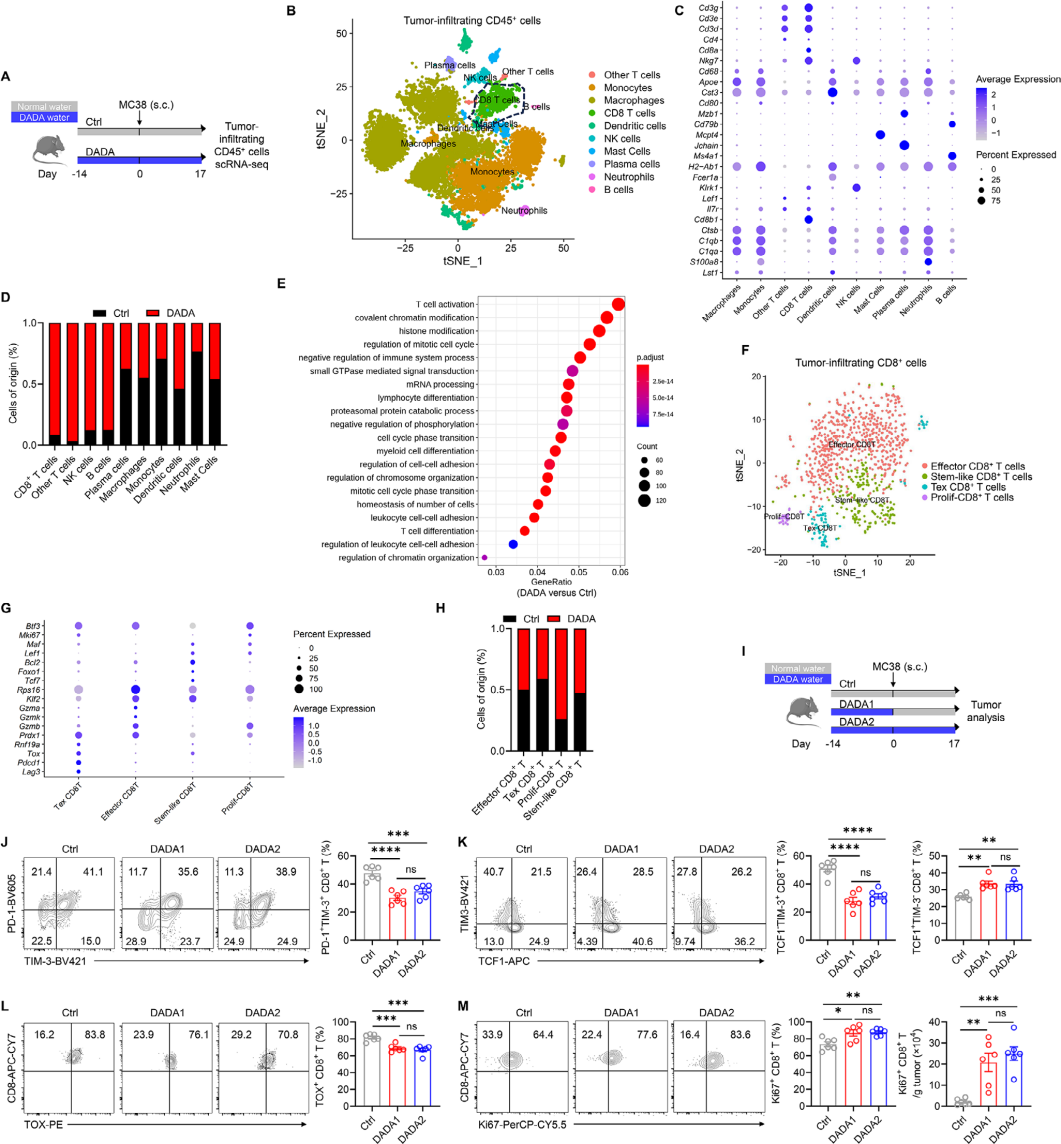
DADA prevents CD8^+^ T cells terminal exhaustion while promoting Tpex cell accumulation in the tumor microenvironment. (A) Schematic experimental procedure for (B–I): WT mice were fed with DADA‐containing or normal water for 31 days and were injected (s.c.) with MC38 cells on day 0. Tumor‐infiltrating CD45^+^ cells were harvested for 10× genomic scRNA‐seq. (B) Biaxial tSNE clustering plots showing tumor‐infiltrating CD45^+^ cells. (C) Dot plot showing the expression of representative genes for each cell type in (B). (D) Relative percentages of each cell type in (B). (E) Top 20 enriched GO terms in CD8^+^ T cells from DADA‐treated mice versus CD8^+^ T cells from control mice. (F) Biaxial tSNE plots showing secondary clusters of CD8^+^ T cells. (G) Dot plot showing the expression of representative genes for each cell subset in (F). (H) Relative percentages of each subset in (F). (I) Schematic experimental procedure for (J–M): WT mice were fed with DADA‐containing or normal water from day ‐14 until the experimental endpoints or until day 0, and were injected (s.c.) with MC38 cells on day 0. (J–M) Representative flow cytometry plots and quantification of PD‐1^+^TIM‐3^+^ (J), TCF1^−^TIM‐3^+^, TCF1^+^TIM‐3^−^ (K), TOX^+^ (L), and Ki67^+^ (M) cells among CD8^+^ T cells from tumor; n = 6. The experiment was repeated three times. Data are presented as mean ± SD and are analyzed by one‐way ANOVA (J–M); *p < 0.05, **p < 0.01, ***p < 0.001, ****p < 0.0001. ns, not significant.

To elucidate the mechanism by which DADA enhances the CD8^+^ T cell anti‐tumor immune responses, we performed single‐cell RNA‐sequencing (scRNA‐seq) on tumor‐infiltrating CD45^+^ T cells isolated from ctrl‐ or DADA‐treated MC38‐bearing mice (Figure 2A). Unbiased clustering analysis showed that these cells could be grouped into ten distinct clusters, encompassing CD8^+^ T cells, other T cells, NK cells, B cells, plasma cells, macrophages, monocytes, dendritic cells, neutrophils, and mast cells (Figure 2B,C; Figure S2A). Remarkably, we found that CD8^+^ T cells, characterized by high expression of *Cd8α*, *Cd8b1*, and *Cd3d*, were present in large proportions among tumor‐infiltrating CD45^+^ T cells and were significantly enriched in the tumor microenvironment of DADA‐treated mice, aligning with the findings from the flow cytometry analysis (Figure 1I and Figure 2B–D). Gene Ontology (GO) analysis showed that DADA treatment led to the upregulation of genes associated with T cell activation, lymphocyte differentiation and T cell differentiation in tumor‐infiltrating CD8^+^ T cells (Figure 2E), implying a potential influence of DADA on the activation and differentiation processes of CD8^+^ T cells within the tumor.

Given that terminal exhaustion differentiation of CD8^+^ T cells induces high expression of inhibitory receptors, impaired production of effector cytokines, low proliferative capacity and diminished persistence, leading to poor responses to tumor antigens [32, 33], we hypothesized that DADA might prevent the terminal exhaustion differentiation of CD8^+^ T cells to exert an anti‐tumor effect. By utilizing specific markers, CD8^+^ T cells were further divided into four subsets: effector CD8^+^ T cells (*Gzma*, *Gzmk* and *Gzmb*), stem‐like CD8^+^ T cells (*Klf2*, *Tcf7*, *Foxo1*, *Bcl2*, and *Lef1*), terminally exhausted CD8^+^ T cells (*Lag3*, *Pdcd1*, and *Tox*) and proliferative CD8^+^ T cells (*Mki67*) (Figure 2F,G; Figure S2B). In mice treated with DADA, there was an upward trend in proliferative CD8^+^ T cells, while terminally exhausted CD8^+^ T cells exhibited a reduction (Figure 2H).

To further validate the effect of DADA on CD8^+^ T cell differentiation, we next performed flow cytometry analysis on tumor‐infiltrating and TDLN CD8^+^ T cells from MC38‐bearing mice (Figure 2I). Both continuous DADA treatment and DADA pretreatment decreased the proportions of PD‐1^+^TIM‐3^+^ and TCF1^−^TIM‐3^+^ Tex subsets, while increasing the proportions of stem‐like TCF1^+^TIM‐3^−^ Tpex subset (Figure 2J,K; Figure S2C). Moreover, decreased expression of TOX, a key transcriptional regulator involved in exhaustion programming and differentiation toward terminal exhaustion, along with increased expression of proliferating marker Ki67, was observed in tumor‐infiltrating and TDLN CD8^+^ T cells from DADA treated‐mice (Figure 2L,M; Figure S2C). The similar observations of reduced Tex cells and increased Tpex cells were also noted in the tumor microenvironment and TDLN in both the B16‐F10 subcutaneous tumor model and B16‐F10 lung metastasis model (Figure S2D–I). Thus, these data collectively suggest that DADA promotes CD8^+^ T cell anti‐tumor immune responses, probably by directing CD8^+^ T cells toward a less terminal exhaustion differentiation and a more stem‐like Tpex differentiation.

### 2.3 DADA Enhances CD8^+^ T Cell Stemness in a PDK1‐Dependent Manner

**FIGURE 3:**
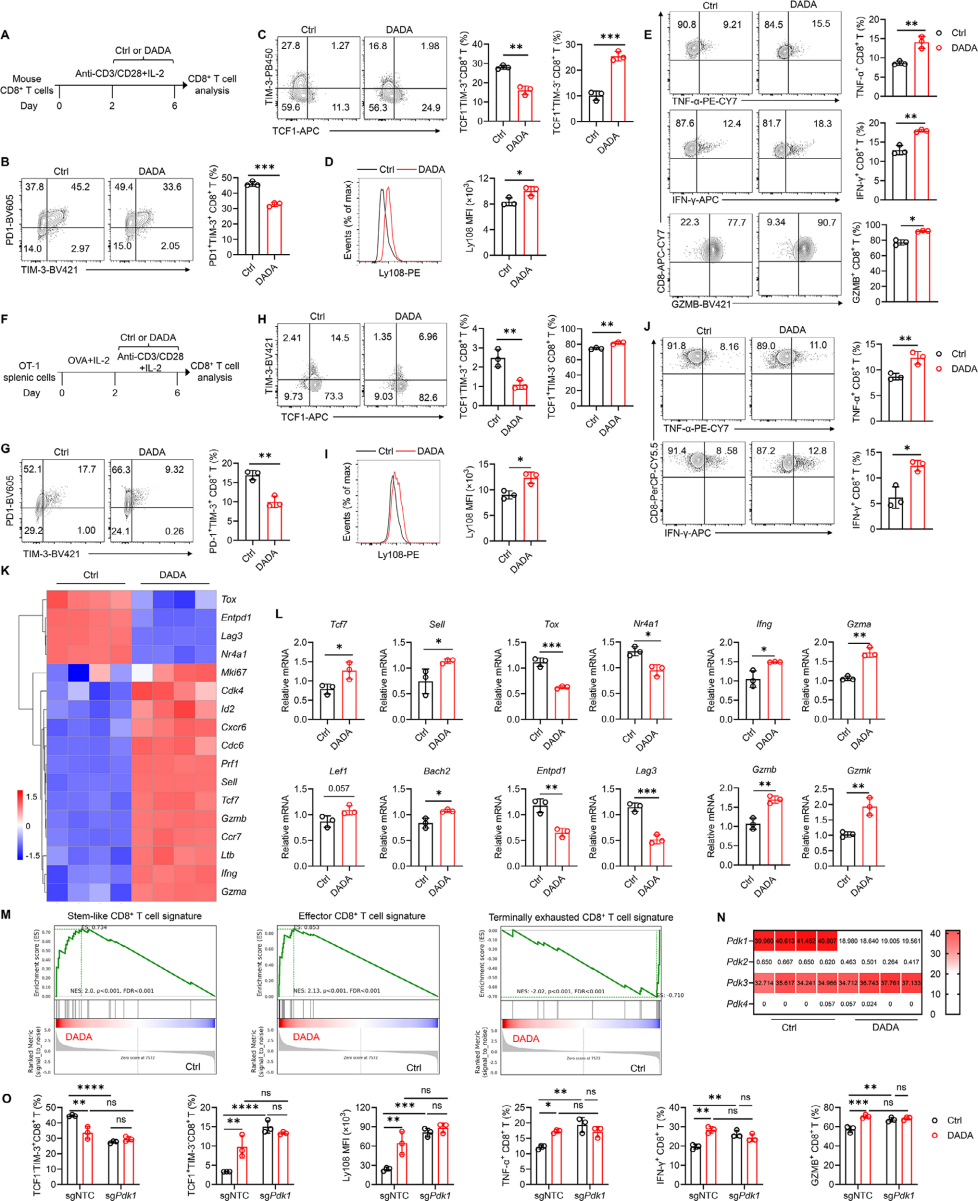
DADA enhances CD8^+^ T cell stemness in a PDK1‐dependent manner. (A) Schematic experimental procedure for the generation of mouse exhausted CD8^+^ T cells for (B–E): purified splenic CD8^+^ T cells were stimulated with anti‐CD3/CD28 mAbs and IL‐2 from for 6 days, with the addition of 40 µM DADA staring from day 3. (B–E) Representative flow cytometry plots and histogram, and quantification of PD‐1^+^TIM‐3^+^ (B), TCF1^−^TIM‐3^+^, TCF1^+^TIM‐3^−^ (C), Ly108 expression (D), TNF‐α^+^, IFN‐γ^+^, and GZMB^+^ (E) cells among CD8^+^ T cells; *n* = 3. The experiment was repeated three times. (F) Schematic experimental procedure for the generation of mouse exhausted OT‐1 CD8^+^ T cells for (G–J): splenic cells from OT‐1 mice were activated with ovalbumin (OVA) peptide and IL‐2 for 2 days, followed by stimulation with anti‐CD3/CD28 mAbs and IL‐2 in the presence of 40 µM DADA from day 3 to day 6. (G–J) Representative flow cytometry plots and histogram, and quantification of PD‐1^+^TIM‐3^+^ (G), TCF1^−^TIM‐3^+^, TCF1^+^TIM‐3^−^ (H), Ly108 expression (I), TNF‐α^+^ and IFN‐γ^+^ (J) cells among CD8^+^ T cells; *n* = 3. The experiment was repeated twice. (K) DADA‐ and DMSO‐treated mouse exhausted CD8^+^ T cells were collected for RNA‐seq. Heatmap showing the expression of selected genes. (L) mRNA levels of the indicated molecules in DADA‐ or DMSO‐treated mouse exhausted CD8^+^ T cells; *n* = 3. The experiment was repeated twice. (M) GSEA of DADA‐ versus DMSO‐treated mouse exhausted CD8^+^ T cells in indicated gene sets. NES, normalized enrichment score. (N) Heatmap showing the expression of *Pdk1*, *Pdk2*, *Pdk3*, and *Pdk4* in DADA‐ and DMSO‐treated mouse exhausted CD8^+^ T cells. (O) Mouse *Pdk1* knockout CD8^+^ T cells induced to exhaustion subjected to DADA treatment were collected. Quantification of TCF1^−^TIM‐3^+^, TCF1^+^TIM‐3^−^, Ly108 expression, TNF‐α^+^, IFN‐γ^+^ and GZMB^+^ cells among CD8^+^ T cells; *n* = 3. Data are presented as mean ± SD and are analyzed by unpaired t test (B‐E, G‐J, and L) and two‐way ANOVA (O); *p < 0.05, **p < 0.01, ***p < 0.001, ****p < 0.0001. ns, not significant.

T cell exhaustion is known to be driven by persistent antigen exposure. To ascertain whether DADA directly regulates CD8^+^ T cell exhaustion differentiation, CD8^+^ T cells were isolated from spleens of WT mice and subjected to continuous stimulation with anti‐CD3/CD28 monoclonal antibodies (mAbs) for 6 days to induce exhaustion differentiation. DADA or the solvent control DMSO was added starting from day 3 to exclude its effect on the early activation of CD8^+^ T cells (Figure 3A). We observed that DADA decreased the frequencies of terminally exhausted PD‐1^+^TIM‐3^+^ and TCF1^−^TIM‐3^+^ subsets while increasing the frequency of stem‐like TCF1^+^TIM‐3^−^ Tpex subset and the expression of stemness‐associated marker Ly108 in a dose‐dependent manner (Figure 3B–D; Figure S3A). This shift toward a more stem‐like and less terminal exhaustion phenotype was accompanied by an elevation in the expression of TNF‐α and IFN‐γ (Figure 3E). Furthermore, a similar effect was observed when DADA was administered continuously from day 0 to day 6 (Figure S3B,C). It is worth noting that DADA did not directly promote the effector function of CD8^+^ T cells; in fact, it suppressed TNF‐α and IFN‐γ expression under conditions where CD8^+^ T cells were stimulated with anti‐CD3/CD28 mAbs for 24 or 48 h (Figure S3D,E). These suggested that the increased production of effector molecules results from the reduced terminal exhaustion of CD8^+^ T cells induced by DADA.

Upon subjecting ovalbumin (OVA)‐specific TCR transgenic OT‐1 cells to persistent TCR stimulation, we observed that DADA enhanced the stem‐like phenotype while decreasing terminal exhaustion in these OT‐1 CD8^+^ T cells (Figure 3F–I). Moreover, DADA treatment led to an increase in the production of TNF‐α and IFN‐γ in OT‐1 CD8^+^ T cells (Figure 3J).

Consistent with the findings obtained from murine T cells, treatment with DADA in vitro led to a higher proportion of the CD62L^+^ cell population, which contains T memory stem cells (Tscm, CD62L^+^CD45RO^−^) and central memory T cells (Tcm, CD62L^−^CD45RO^+^), among human peripheral blood CD8^+^ T cells (Figure S3F,G). Additionally, these CD8^+^ T cells displayed increased expression of stemness‐associated marker CCR7, effector molecules GZMB and IFN‐γ, along with reduced expression of terminally exhausted markers TIM‐3 and TIGIT (Figure S3H–J).

To better characterize the differentiation status of CD8^+^ T cells, we performed bulk RNA sequencing (RNA‐seq) analysis on DMSO‐ or DADA‐treated mouse CD8^+^ T cells, and observed that DADA treatment led to a distinct transcriptional profile. Genes related to cell stemness, such as *Tcf7*, *Sell*, *Ccr7*, and *Id2*, along with genes related to effector functions, such as *Ifng*, *Gzma* and *Gzmk*, were enriched in DADA‐treated CD8^+^ T cells (Figure 3K). Conversely, genes associated with terminally exhaustion, such as *Tox*, *Nr4a1*, *Endpd1*, and *Lag3*, were decreased in DADA‐treated CD8^+^ T cells (Figure 3K). Quantitative polymerase chain reaction (qPCR) analysis further validated increased mRNA expression of genes related to cell stemness, effector functions, and terminally exhaustion in CD8^+^ T cells following DADA treatment (Figure 3L). Gene set enrichment analysis (GSEA) demonstrated a higher enrichment of both a stem‐like signature and an effector CD8^+^ T signature, along with a decreased terminally exhausted signature, in DADA‐treated CD8^+^ T cells (Figure 3M). Together, these findings suggested that DADA prevents the differentiation of CD8^+^ T cell into terminally exhausted states, preserves their stemness and maintains their capacity to produce cytokines.

DADA is known as an inhibitor of PDK, whose family comprises four distinct isozymes—PDK1, PDK2, PDK3, and PDK4 [34]. We therefore ought to investigate which of these PDK isoenzymes regulate CD8^+^ T cell stemness. RNA‐seq data revealed high expression levels of *Pdk1* and *Pdk3* in CD8^+^ T cells undergoing persistent TCR stimulation, with *Pdk1* being downregulated following DADA treatment (Figure 3N). This indicated that PDK1 might play a crucial role in the differentiation of CD8^+^ T cells and could be a potential target of DADA. Accordingly, using single‐guide RNA (sgRNA) to silence *Pdk1* was sufficient to inhibit terminal exhaustion, and enhance the stem‐like characteristics and cytokine production of these cells to a level comparable to that induced by DADA (Figure 3O). Moreover, DADA was unable to further enhance the cell stemness and cytokine production in *Pdk1* knockout CD8^+^ T cells (Figure 3O; Figure S3K). Furthermore, the overexpression of *Pdk1* increased the frequency of Tex subset while reducing the frequencies of Tpex subset and effector molecule‐expressing cells, with these changes being reversible by DADA (Figure S3L,M). In contrast, the silencing of *Pdk2*, *Pdk3*, or *Pdk4* did not affect the terminal exhaustion, cell stemness or cytokine production in CD8^+^ T cells, irrespective of the presence of DADA (Figure S3N,O), underscoring the specific and non‐redundant role of PDK1 in mediating DADA's promotion of CD8^+^ T cell stemness. These results collectively suggest that DADA enhances CD8^+^ T cell stemness in a PDK1‐dependent manner.

### 2.4 DADA‐Triggered Metabolic Reprogramming Promotes CD8^+^ T Cell Stemness

**FIGURE 4:**
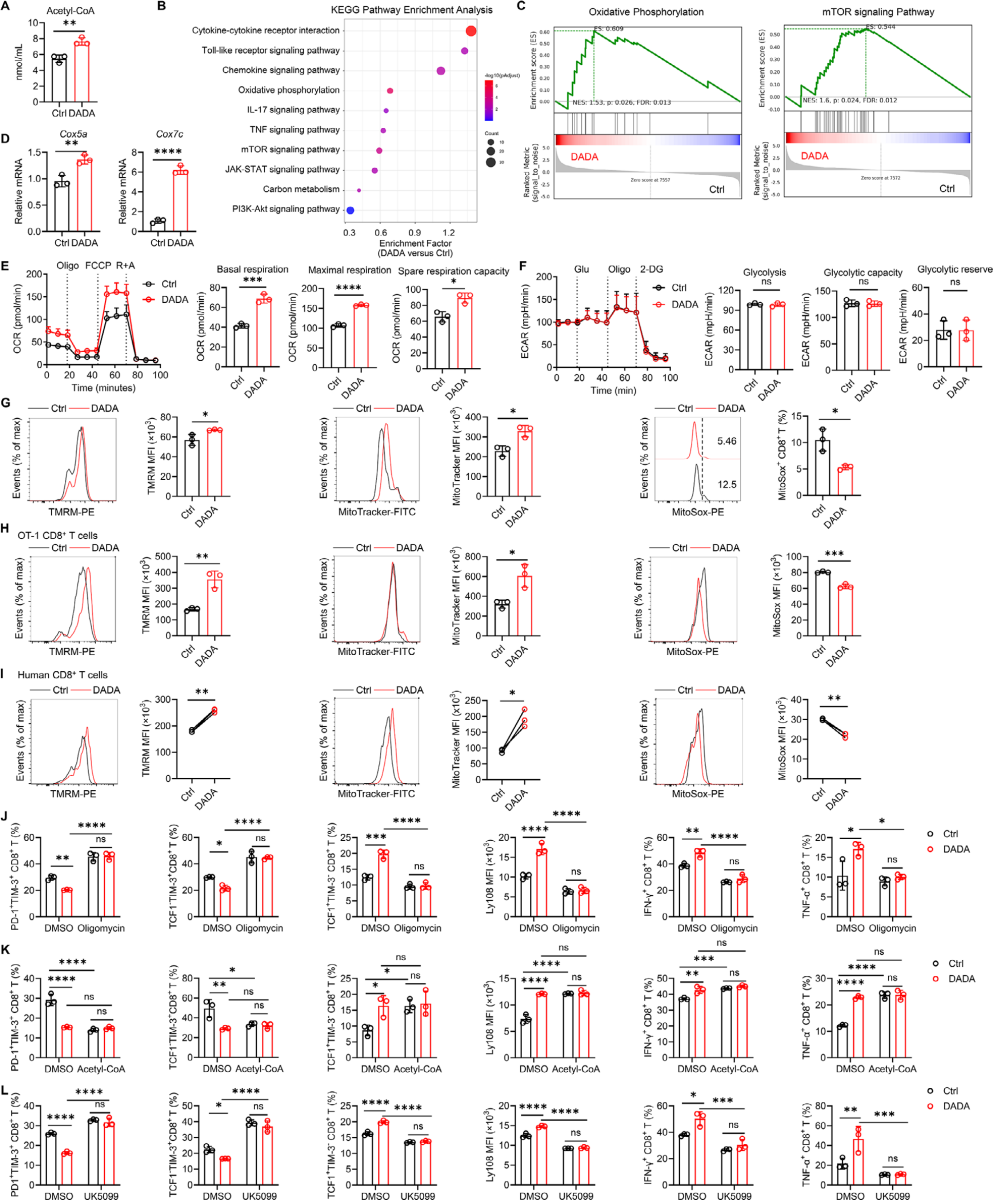
DADA enhances CD8^+^ T cell stemness by triggering OXPHOS. (A) Acetyl‐CoA contents in DADA‐ and DMSO‐treated mouse exhausted CD8^+^ T cells; *n* = 3. The experiment was repeated twice. (B) Top 10 pathways upregulated in DADA‐ versus DMSO‐treated exhausted CD8^+^ T cells. (C) GSEA of DADA‐ versus DMSO‐treated mouse exhausted CD8^+^ T cells in indicated gene sets. (D) mRNA levels of the indicated molecules in DADA‐ and DMSO‐treated mouse exhausted CD8^+^ T cells; *n* = 3. The experiment was repeated twice. (E) OCR of DADA‐ and DMSO‐treated mouse exhausted CD8^+^ T cells. Oligo, oligomycin; FCCP, carbonyl cyanide p‐trifluoromethoxyphenylhydrazone; R+A, rotenone and antimycin A; *n* = 3. The experiment was repeated twice. (F) Extracellular acidification rate (ECAR) of DADA‐ and DMSO‐treated mouse exhausted CD8^+^ T cells. Glu, glucose; Oligo, oligomycin; 2‐DG, 2‐deoxyglucose; *n* = 3. The experiment was repeated twice. (G) The mitochondrial membrane potential (TMRM staining), mitochondrial mass (MitoTracker Deep Red) and mitochondrial superoxide (MitoSox Red) of DADA‐ and DMSO‐treated mouse exhausted CD8^+^ T cells were measured. Representative histograms and quantification of TMRM, MitoTracker, and MitoSox in CD8^+^ T cells; *n* = 3. The experiment was repeated three times. (H) Representative histograms and quantification of TMRM, MitoTracker, and MitoSox in DADA‐ and DMSO‐treated mouse exhausted OT‐1 CD8^+^ T cells; *n* = 3. The experiment was repeated three times. (I) Representative histograms and quantification of TMRM, MitoTracker, and MitoSox in DADA‐ and DMSO‐treated human exhausted CD8^+^ T cells; *n* = 3. The experiment was repeated three times. (J) Mouse exhausted CD8^+^ T cells subjected to DADA and Oligomycin (1 µM) combined treatment or single treatment were collected. Quantification of PD‐1^+^TIM‐3^+^, TCF1^−^TIM‐3^+^, TCF1^+^TIM‐3^−^, Ly108 expression, TNF‐α^+^ and IFN‐γ^+^ cells among CD8^+^ T cells; *n* = 3. The experiment was repeated twice. (K) Mouse exhausted CD8^+^ T cells subjected to DADA and Acetyl‐CoA (2 mM) combined treatment or single treatment were collected. Quantification of PD‐1^+^TIM‐3^+^, TCF1^−^TIM‐3^+^, TCF1^+^TIM‐3^−^, Ly108 expression, TNF‐α^+^ and IFN‐γ^+^ cells among CD8^+^ T cells; *n* = 3. The experiment was repeated twice. (L) Mouse exhausted CD8^+^ T cells subjected to DADA and UK5099 (20 µM) combined treatment or single treatment were collected. Quantification of PD‐1^+^TIM‐3^+^, TCF1^−^TIM‐3^+^, TCF1^+^TIM‐3^−^, Ly108 expression, TNF‐α^+^ and IFN‐γ^+^ cells among CD8^+^ T cells; *n* = 3. The experiment was repeated twice. Data are presented as mean ± SD and are analyzed by unpaired t test (A, D, and E–H), paired t test (I) and two‐way ANOVA (J–L); *p < 0.05, **p < 0.01, ***p < 0.001, ****p < 0.0001. ns, not significant.

PDC, which catalyzes the irreversible conversion of pyruvate to Acetyl‐CoA in the mitochondria, plays a critical role in maintaining the delicate balance between glycolysis and OXPHOS, with its enzymatic activity inhibited by PDK‐mediated phosphorylation and activated by pyruvate dehydrogenase phosphatase‐mediated dephosphorylation [28]. Given our observation that DADA enhances CD8^+^ T cell stemness through PDK1, we proceeded with further investigations into its effect on CD8^+^ T cell metabolism. We indeed detected DADA treatment led to higher concentrations of Acetyl‐CoA in CD8^+^ T cells (Figure 4A). As Acetyl‐CoA can provide acetyl group onto histones to facilitate histone acetylation, we consistently observed increased levels of histone H3 lysine residue 27 acetylation (H3K27ac)—a recognized epigenetic modification known to enhance the stemness of T cells—in DADA‐treated CD8^+^ T cells (Figure S4A). Pathway enrichment and GSEA analyses revealed an enrichment of metabolic pathways, including OXPHOS, the mTOR signaling pathway, and the carbon metabolism in DADA‐treated CD8^+^ T cells (Figure 4B,C). qPCR analysis confirmed the increased mRNA expression of OXPHOS‐related genes such as *Cox5a* and *Cox7c* (Figure 4D). Seahorse analysis further validated that DADA treatment promoted OXPHOS in CD8^+^ T cells, as measured by oxygen consumption rate (OCR), in terms of basal respiration, maximal respiration and spare respiratory capacity (Figure 4E). Notably, DADA exhibited a negligible effect on glycolysis in these cells (Figure 4F).

Mitochondria serve as hubs of metabolic activity that couple the TCA cycle to OXPHOS for optimal energy production [17]. We consistently observed that DADA improved mitochondrial fitness, as evidenced by the increased mitochondrial membrane potential and mass, as well as decreased mitochondrial ROS production (Figure 4G). Similar improvements in metabolic fitness induced by DADA were also noted in OT‐1 CD8^+^ T cells and human CD8^+^ T cells (Figure 4H,I). These results collectively suggested that DADA orchestrates a metabolic shift toward enhanced OXPHOS and improved mitochondrial fitness in CD8^+^ T cells.

We next investigated whether the enhanced OXPHOS induced by DADA contributes to the promotion of CD8^+^ T cell stemness. CD8^+^ T cells were exposed to persistent TCR stimulation in the presence of DADA with or without oligomycin (an OXPHOS pan inhibitor). The data showed that when OXPHOS was blocked, DADA failed to decrease terminal exhaustion, enhance cell stemness and improve mitochondrial fitness in CD8^+^ T cells (Figure 4J; Figure S4B).

The increased OXPHOS is supported by the increased TCA cycle. Accordingly, directly feeding CD8^+^ T cells with Acetyl‐CoA, a substrate that fuels the TCA cycle, replicated DADA's promotion on cell stemness and mitochondria fitness (Figure 4K; Figure S4C). Moreover, the simultaneous administration of DADA and Acetyl‐CoA did not lead to a better improvement (Figure 4K; Figure S4C). Given that cytosolic pyruvate needs to be transported to the mitochondria, a process mediated by the mitochondrial pyruvate carrier (MPC) [35], for subsequent conversion into acetyl‐CoA by PDC, we utilized UK5099 to block MPC activity. The blockade of MPC abolished the effects of DADA on CD8^+^ T cells (Figure 4L; Figure S4D). Together, these findings suggest that DADA enhances OXPHOS and mitochondria fitness by boosting intracellular Acetyl‐CoA production, thereby promoting the stemness of CD8^+^ T cells.

### 2.5 DADA Improves ACT and ICB Immunotherapy Efficacy

**FIGURE 5:**
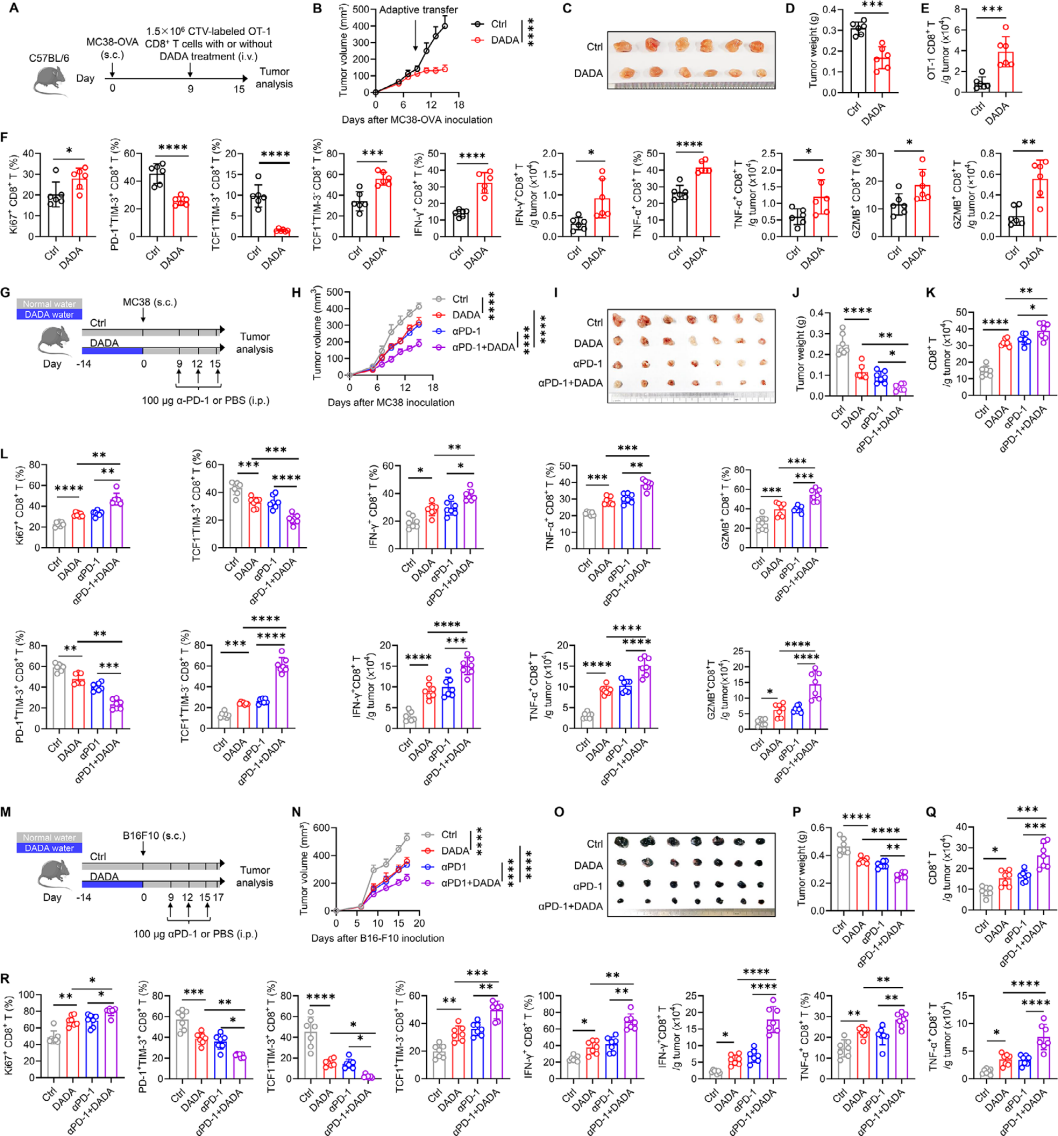
DADA improves the efficacy of ACT and ICB immunotherapies. (A) Schematic experimental procedure in (B–F): CTV‐labeled OT‐1 cells, which had undergone an initial activation with OVA peptide for 2 days and subsequent stimulation with anti‐CD3/CD28 mAbs and IL‐2 in the presence of DADA for 4 days, were transferred into MC38‐OVA tumor‐bearing mice. (B–D) Tumor growth curves (B), images of tumors (C), and tumor weights (D) at 15 days after MC38‐OVA inoculation; *n* = 6. (E,F) Quantification of transferred OT‐1 CD8^+^ T (E), Ki67^+^ CD8^+^ T, PD‐1^+^TIM‐3^+^ CD8^+^ T, TCF1^−^TIM‐3^+^ CD8^+^ T, TCF1^+^TIM‐3^−^ CD8^+^ T, IFN‐γ^+^ CD8^+^ T, TNF‐α^+^ CD8^+^ T, and GZMB^+^ CD8^+^ T (F) cells from tumor; *n* = 6. (G) Schematic experimental procedure in (H–L): WT mice were fed with DADA‐containing or normal water from day ‐14 until day 0, and were injected (s.c.) with MC38 cells on day 0. Anti‐PD‐1 mAb were injected (i.p.) on day 9, 12 and 15. (H–J) Tumor growth curves (H), images of tumors (I), and tumor weights (J) at 15 days after MC38 inoculation; *n* = 7. The experiment was repeated three times. (K,L) Quantification of CD8^+^ T (K), Ki67^+^ CD8^+^ T, PD‐1^+^TIM‐3^+^ CD8^+^ T, TCF1^−^TIM‐3^+^ CD8^+^ T, TCF1^+^TIM‐3^−^ CD8^+^ T, IFN‐γ^+^ CD8^+^ T, TNF‐α^+^ CD8^+^ T, and GZMB^+^CD8^+^ T (L) cells from tumor; *n* = 7. The experiment was repeated three times. (M) Schematic experimental procedure in (N–R): WT mice were fed with DADA‐containing or normal water from day ‐14 until day 0, and were injected (s.c.) with B16‐F10 cells on day 0. Anti‐PD‐1 mAb were injected (i.p.) on day 9, 12 and 15. (N‐P) Tumor growth curves (N), images of tumors (O), and tumor weights (P) at 17 days after B16‐F10 inoculation; *n* = 7. The experiment was repeated three times. (Q,R) Quantification of CD8^+^ T (Q), Ki67^+^ CD8^+^ T, PD‐1^+^TIM‐3^+^ CD8^+^ T, TCF1^−^TIM‐3^+^ CD8^+^ T, TCF1^+^TIM‐3^−^ CD8^+^ T, IFN‐γ^+^ CD8^+^ T, and TNF‐α^+^ CD8^+^ T (R)cells from tumor; *n* = 7. The experiment was repeated three times. Data are presented as mean ± SD and are analyzed by two‐way ANOVA (B, H, and N), unpaired t test (D–F), and one‐way ANOVA (J–L, and P‐R); *p < 0.05, **p < 0.01, ***p < 0.001, ****p < 0.0001. ns, not significant.

Recent studies have highlighted that stem‐like Tpex cells as the key T cell subset that responds to ACT therapy and ICB therapies [2, 3, 4, 13, 14, 15, 16]. We investigated whether CD8^+^ T cells treated with DADA exhibited better anti‐tumor responses post‐transfer. Splenic cells isolated from OT‐1 mice, following initial OVA peptide activation, were cultured with anti‐CD3/CD28 mAbs in the presence of DADA or DMSO. These cells were then labeled with CTV and adoptively transferred into MC38‐OVA‐bearing mice (Figure 5A). Compared to control OT‐1 cells, DADA‐treated OT‐1 cells demonstrated a significant inhibition of tumor growth (Figure 5B–D). Moreover, we detected increased cell counts of transferred OT‐1 CD8^+^ T cells, accompanied by elevated Ki67 expression in these cells, in the tumor microenvironment of mice receiving DADA‐treated OT‐1 cells, suggesting improved persistence and proliferation of DADA‐treated OT‐1 cells in vivo (Figure 5E,F). Additionally, DADA‐treated OT‐1 CD8^+^ T cells within tumor and TDLN exhibited a shift toward a less Tex and more Tpex phenotype, with enhanced effector functions (Figure 5F; Figure S5A).

We next tested the influence of DADA on ICB immunotherapy. In subcutaneous tumor models characterized by either strong immunogenicity (MC38 and B16‐F10 models) or weak immunogenicity (4T1 and LLC models), the administration of DADA effectively enhanced the efficacy of anti‐PD‐1 therapy, and increased the cell counts of tumor‐infiltrating CD8^+^ T cells and their Ki67 expression (Figure 5G–R; Figure S5B–K). Reduced Tex cells, increased Tpex cells and higher proportions of effector cytokine‐producing CD8^+^ T cells were detected within the tumor and TDLN in mice receiving anti‐PD‐1 antibody combined with DADA (Figure 5L,R; Figure S5L,M). These results collectively suggested that DADA bolsters the Tpex population and improves the efficacy of ACT and ICB immunotherapies.

### 2.6 DADA Potentiates the Anti‐Tumor Potency of Human CAR‐T Cells

**FIGURE 6:**
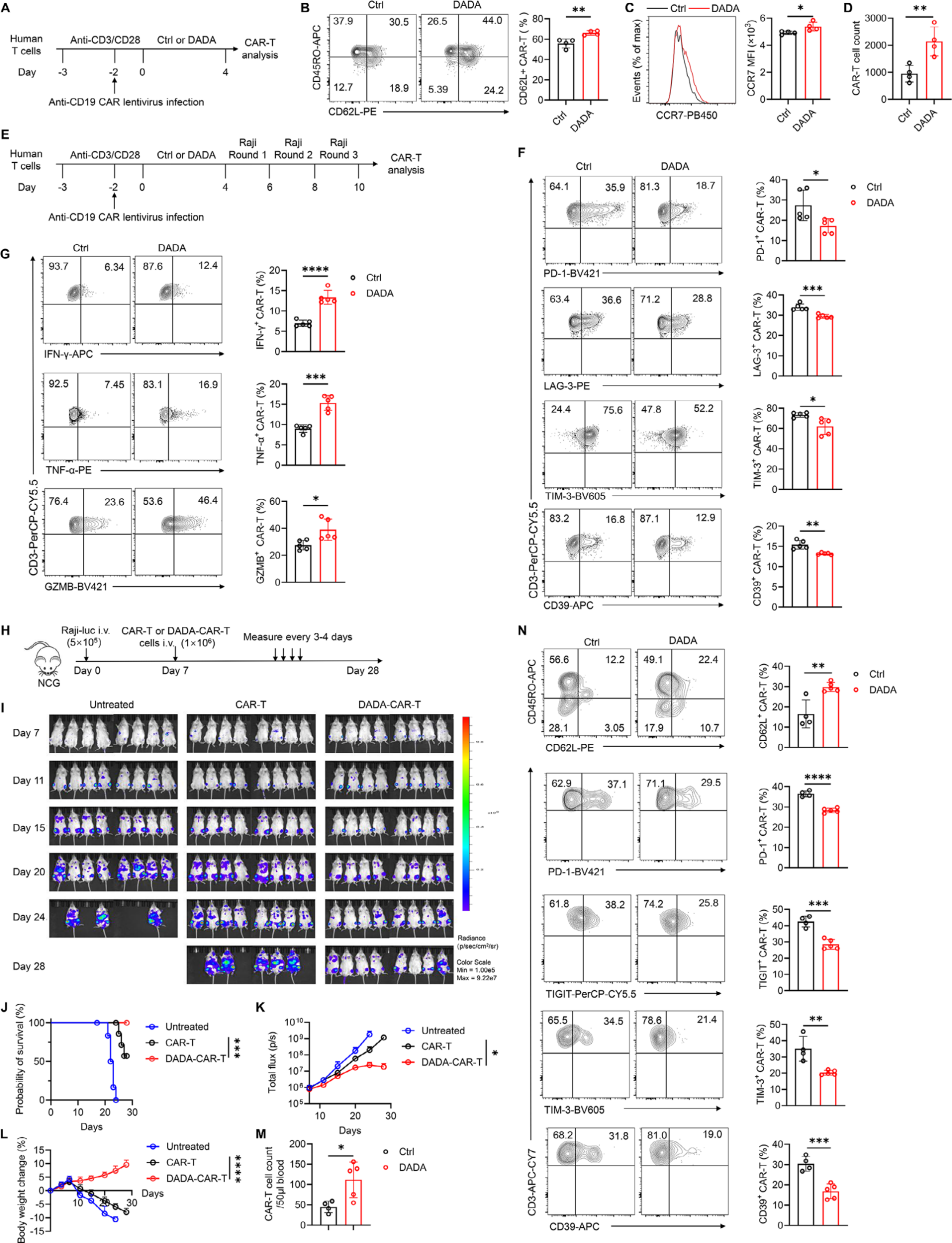
DADA potentiates the anti‐tumor potency of human CAR‐T cells. (A) Schematic experimental procedure for (B–D): CAR‐T cells were generated and were then treated with DADA (40 µM) for 4 days. (B,C) Representative flow cytometry plots, histogram, and quantification of CD62L^+^ cell and the CCR7 expression among CAR‐T cells; *n* = 4. (D) Quantification of total CAR‐T cells; *n* = 4. (E) Schematic experimental procedure for (F) and (G): CAR‐T cells were generated and were then treated with DADA (40 µM) for 4 days. Subsequently, these CAR‐T cells were collected, washed to remove DADA, and underwent 3 rounds of co‐culture with Raji cells at an effector‐to‐target ratio of 1:1. DADA was not present during the Raji cell stimulation phase. (F,G) Representative flow cytometry plots and quantification of PD‐1^+^, LAG‐3^+^, TIM‐3^+^, and CD39^+^ (F), IFN‐γ^+^, TNF‐α^+^ and GZMB^+^ (G) cells among CAR‐T cells; *n* = 5. (H) Schematic experimental procedure for (I)‐(N): NCG mice were injected (i.v.) with Raji‐luc cells on day 0, and were injected (i.v.) with DADA‐ or DMSO‐pretreated CAR‐T cells on day 7. (I–L) Bioluminescence images (I), survival curves (J), average bioluminescence curves (K), weight loss curves (L) were shown; n = 8. (M,N) Representative flow cytometry plots and quantification of CAR‐T cells (M), CD62L^+^, PD‐1^+^ TIM‐3^+^, TIGIT^+^, and CD39^+^, cells among CAR‐T cells (N) from peripheral blood on day 28. Data are presented as mean ± SD and are analyzed by unpaired t test (B–D, F, G, M, and N), log‐rank Mantel–Cox test (J), and two‐way ANOVA (K and L); *p < 0.05, **p < 0.01, ***p < 0.001, ****p < 0.0001. ns, not significant.

To gain further insight into potential therapeutic strategies, we investigated the impact of DADA on genetically engineered chimeric antigen receptor (CAR)‐T cells. Second‐generation CAR‐T cells targeting CD19 were generated through lentivirus infection and subsequently exposed to either DADA or DMSO (Figure 6A), and CAR expression was quantified using GFP as a reporter (Figure S6A,B). The DADA treatment increased the proportion of the CD62L^+^ cell population among CAR‐T cells, including both CD4^+^ and CD8^+^ subsets (Figure 6B; Figure S6C–E). Moreover, DADA increased the expression of CCR7 on CAR‐T cells and promoted the expansion of CAR‐T cells in vitro (Figure 6C,D). These findings demonstrated that DADA endows CAR‐T cells with a stem‐like phenotype.

Next, DADA‐ or DMSO‐treated CAR‐T cells were subjected to multiple rounds of stimulation with Raji cells which express CD19 to induce exhaustion (Figure 6E). Compared to DMSO‐treated CAR‐T cells, DADA treatment led to a reduction in the expression of PD‐1, LAG‐3, TIM‐3, and CD39, while increasing the expression of IFN‐γ, TNF‐α and GZMB, including both CD4^+^ and CD8^+^ subsets (Figure 6F,G; Figure S6F,G). Moreover, consistent alterations were also observed in CAR‐T cells when they were generated from purified human CD8^+^ T cells (Figure S6H,I). These finding suggest that DADA prevents the terminal exhaustion of CAR‐T cells over persistent antigen stimulation.

We then evaluated the anti‐tumor efficacy of DADA‐treated CD19 CAR‐T cells using a Raji xenograft model. Immune‐deficient NCG (NOD/ShiLtJGpt‐*Prkdc* ^em26Cd52^ *Il2rg* ^em26Cd22^/Gpt) mice were intravenously injected with Raji cells stably expressing luciferase (Raji‐luc), and then received the adoptive transfer of a suboptimal number of DMSO‐ or DADA‐treated CAR‐T cells (Figure 6H). The infusion of DADA‐treated CAR‐T cells led to improved tumor control (Figure 6I–L). Moreover, higher cell counts of CAR‐T cells were detected in the blood of mice receiving DADA‐treated CAR‐T cells (Figure 6M). We also found that DADA‐treated CAR‐T cells displayed lower expression of exhaustion markers in vivo (Figure 6N). Importantly, no tissue damage was observed in mice received DADA‐treated CAR‐T cells (Figure S6J). These results suggested that DADA‐treated CAR‐T cells demonstrated a better anti‐tumor potency, prolonged in vivo persistence/expansion and reduced terminal exhaustion. Collectively, these findings underscore the potential of DADA as a therapeutic strategy to improve the efficacy of CAR‐T cell therapy.

## 3 Discussion

The terminal exhaustion of CD8^+^ T cells poses a significant challenge to effective anti‐tumor immune responses and limits the efficacy of many current immunotherapies. In contrast, stem‐like Tpex cells emerge as a promising subset recognized for their capacity to generate enduring anti‐tumor immune responses and exhibit heightened responsiveness to immunotherapies [2, 3, 4, 12, 13, 14, 15, 16]. Our study sheds light on the potential of DADA in enhancing CD8^+^ T cell‐mediated anti‐tumor immunity and enhances the effectiveness of ICB and ACT immunotherapies by bolstering the Tpex population.

PDC, whose enzymatic activity is inhibited by PDK‐mediated phosphorylation, functions as the key enzyme governing Acetyl‐CoA production. Inhibition of PDK by DADA led to increased acetyl‐CoA production. Apart from its role as a substrate of TCA cycle, Acetyl‐CoA can provide acetyl group onto histones to facilitate histone acetylation, an epigenetic modification known to bolster CAR‐T cell resistance to exhaustion and to enhance T cell stemness [36, 37]. The present study has demonstrated the contribution of DADA‐induced elevation of Acetyl‐CoA and OXPHOS to the enhancement of cellular stemness; however, the comprehensive impact of DADA on histone acetylation in CD8^+^ T cells remains unexplored. Given the prolonged persistence and durable anti‐tumor responses observed in DADA‐treated OT‐1 and CAR‐T cells in vivo, it is reasonable to speculate that the long‐lasting effects of DADA on T cell stemness may involve epigenetic modifications, particularly histone acetylation. It will be worth investigating the interrelationships among DADA, histone acetylation and CD8^+^ T cell stemness in future.

Tumor cells predominantly rely on heightened glycolysis and lactic acid fermentation to sustain their rapid proliferation and growth. DADA exhibits anti‐cancer capabilities by inhibiting glycolysis in tumor cells [30, 31]. Moreover, inhibition of glycolysis by DADA also mitigates lactic acid accumulation, a factor known to impair the effector functions of CD8^+^ T cells [38, 39, 40]. Here, our study reveals a previously unknown aspect by illustrating that DADA can directly influence CD8^+^ T cells, enhancing their stemness and fostering robust anti‐tumor immune responses. This deepens our understanding of the anti‐tumor mechanisms of DADA, indicating that utilizing DADA for tumor treatment could address multiple objectives simultaneously, including restraining tumor cell growth, ameliorating the acidic tumor microenvironment, and enhancing CD8^+^ T cell stemness. These findings hint at the promising clinical potential of DADA in tumor therapy.

It is noteworthy that we observed DADA significantly improving CD8^+^ T cell OXPHOS while exerting a minimal influence on glycolysis. The different effect of DADA on glycolysis in CD8^+^ T cells compared to tumor cells highlights the intricacy of metabolic processes in distinct cellular contexts. We speculate that within the context of CD8^+^ T cell exhaustion, the pivotal factor influencing lactic acid production may be the expression level or enzymatic activity of lactate dehydrogenase (LDH), which is responsible for converting pyruvate to lactic acid, rather than cellular pyruvate levels. Although lactic acid has been linked to the modulation of CD8^+^ T cell stemness [41], the specific role of LDH in CD8^+^ T cell stemness remains a topic requiring further exploration.

Mitochondrial dysfunction characterized by membrane depolarization, metabolic alterations, and excessive ROS generation, promotes the transition from Tpex cells to Tex cells through glycolytic reprogramming [23, 24, 25, 42, 43]. A recent study has revealed that inhibiting glycolysis through pharmacological inhibition of hexokinase or through genetic ablation of *Hif1α* prevents the transition from Tpex cells to Tex cells [42]. However, these two approaches not only inhibit glycolysis, but also induce metabolic reprogramming, such as increased OCR and mitochondrial respiration, raising questions about which factor truly governs T cell stemness and terminal exhaustion.

Given the observed specific effect of DADA on CD8^+^ T cell OXPHOS, we found that improving OXPHOS and mitochondrial fitness without perturbing glycolysis is sufficient to preserve CD8^+^ T cell stemness. This implies that glycolytic shift might be a consequence of metabolic reprogramming rather than being the primary driver of inducing CD8^+^ T cell stemness. Enhancing OXPHOS and mitochondrial fitness could be pivotal in promoting Tpex cells. Future investigations focusing on enhancing OXPHOS and mitochondrial fitness, exemplified by DADA, hold significant promise for improving the effectiveness of CD8^+^ T cell‐based cancer immunotherapies.

Recent studies have highlighted metabolic interventions, such as MPC inhibitors and isocitrate dehydrogenase 2 (IDH2) inhibitors, as pivotal regulators of CD8^+^ T cell fate. MPC inhibitors block cytosolic pyruvate import into mitochondria, thereby limiting acetyl‐CoA production; this mechanistic feature drives functional incompatibility with DADA. Consistently, MPC deficiency has been reported to enhance terminal exhaustion of CD8^+^ T cells and impair their effector functions in the tumor microenvironment [44, 45]. In contrast, IDH2 inhibitors act on the TCA cycle by inhibiting the conversion of isocitrate to α‐ketoglutarate, modulating metabolic intermediates and redox homeostasis to prevent T cell exhaustion [46]. Given their non‐overlapping mechanism with DADA, these inhibitors hold promising synergistic potential via complementary effects. These mechanistic insights into metabolic interventions provide a rational foundation for developing combinatorial strategies to enhance CD8^+^ T cell stemness and anti‐tumor efficacy.

Currently, CAR‐T cells designed to target specific tumor types, such as B cell malignancies, have shown remarkable efficacy in both experimental and clinical settings [47, 48]. However, CAR‐T therapy imposes considerable economic burden on patients due to the high cost of CAR‐T cell manufacture and logistical complexities [49]. Moreover, the intricate CAR‐T cell manufacturing process generally encompasses isolation and enrichment of T cells, activation and expansion of T cells, transduction of T cells with CAR, and ex vivo expansion of CAR‐T cells, demanding a lot of time. Any delays in CAR‐T cell manufacture could prove fatal for this highly malignant disease [50]. Thus, a strategy involving a low but effective cell dosage could help reduce the manufacturing cost and shorten the manufacture‐to‐infusion timeline [51]. Notably, DADA‐treated CAR‐T cells at a sub‐optimal dose exhibited a superior anti‐tumor potency, prolonged persistence, and resistance to exhaustion in vivo, offering a promising direction for improving accessibility and timely delivery of CAR‐T cell therapy [52, 53].

## 4 Methods

### 4.1 Mice

Male C57BL/6J mice (6–8 weeks old), female BALB/c mice (6–8 weeks old), and male NCG mice (6–8 weeks old) were purchased from GemPharmatech (Nanjing, China). Male OT‐1 mice (6‐8 weeks old) were purchased from Shanghai Model Organisms Center (Shanghai, China). All mice were housed in specific pathogen‐free conditions, and were maintained at a temperature of 22–25°C, a humidity of 55%, and a 12 h light‐dark cycle, with free access to food and water. All animal experiments were conducted in accordance with procedures approved by the Institutional Animal Care and Use Committee of Anhui Medical University and IHM (approval number: IHM‐AP‐2025‐177).

### 4.2 Human Peripheral Blood Mononuclear Cells

All human samples used in this study were approved by the Ethics Committee of Anhui Medical University (approval number: 81250540). Peripheral blood samples were collected from healthy volunteers. All experimental procedures were conducted in strict compliance with the principles of the Declaration of Helsinki and its subsequent revisions. Written informed consent was obtained from all participants.

### 4.3 Cell Lines

The mouse melanoma B16‐F10 cell line was purchased from the Cell Bank of the Chinese Academy of Sciences (Shanghai, China). The colon cancer MC38 cell line was purchased from the National Bio‐Technology Collection Center (BNCC, Beijing, China). MC38‐OVA cells were made by lentiviral transduction of OVA gene into MC38 cells. Raji and Raji‐luc cells were purchased from the Cell Culture Center of the Chinese Academy of Medical Sciences (Beijing, China). All cells were tested for mycoplasma contamination using the Myco‐Off Mycoplasma Clearance Reagent (Cat#D103‐01, Vazyme, China) and confirmed to be free of mycoplasma. B16‐F10, MC38, and MC38‐OVA cells were cultured in high‐glucose DMEM (Cat#11965092, Gibco, USA) supplemented with 10% fetal bovine serum (Cat#10099158, Gibco, USA) and 1% penicillin/streptomycin (Cat#10378016, Gibco, USA). Raji and Raji‐luc cells were cultured in RPMI‐1640 (Cat#A4192301, Gibco, USA) supplemented with 10% fetal bovine serum and 1% penicillin/streptomycin. All cells were maintained in a 5% CO_2_ incubator at 37°C.

### 4.4 Antibodies

For murine T cells, anti‐mouse CD45.2‐FITC (clone 104; Cat#109806), anti‐mouse CD45.2‐APC‐CY7 (Clone 104; Cat#109824), anti‐mouse CD3‐FITC (Clone 17A2; Cat#100204), anti‐mouse CD4‐PerCP‐CY5.5 (Clone GK1.5; Cat#100434), anti‐mouse CD8‐PerCP‐CY5.5 (Clone 53–6.7; Cat#100734), anti‐mouse CD8α‐FITC (Clone 53–6.7; Cat#100706), anti‐mouse CD8β‐FITC (Clone YTS156.7.7; Cat#126606), anti‐mouse CD8α‐APC‐CY7 (Clone 53–6.7; Cat#557654), anti‐mouse PD‐1‐BV605 (Clone 29F.1A12; Cat#135220), anti‐mouse TIM‐3‐BV421 (Clone RMT3‐23; Cat#119723), anti‐mouse Ly108‐PE (Clone 330‐AJ; Cat#134606), anti‐mouse TCF‐1‐APC (Clone 7F11A10; Cat#655210), anti‐mouse TOX‐PE (Clone TXRX10; Cat#144506), anti‐mouse Ki67‐PerCP‐CY5.5 (Clone 11F6; Cat#50‐237‐5907), anti‐mouse IFN‐γ‐APC (Clone XMG1.2; Cat#505810), anti‐mouse IFN‐γ‐PE (Clone XMG1.2; Cat#05808), anti‐mouse/human GZMB‐BV421 (Clone GB11; Cat#563389); anti‐mouse/human GZMB‐FITC (Clone QA18A28; Cat#396404), anti‐mouse TNF‐α‐PE‐CY7 (Clone MP6‐XT22; Cat#506324) were purchased from BioLegend. For human T cells, anti‐human CD3‐APC‐CY7 (Clone HIT3a; Cat#300318), anti‐human CD8‐PE‐CY7 (Clone SK1; Cat#344711), anti‐human CD45RO‐APC (Clone UCHL1; Cat#376809), anti‐human CD45RA‐PerCP‐CY5.5 (Clone HI100; Cat#B358423), anti‐human CD62L‐PE (Clone DREG‐56; Cat#304805), anti‐human CCR7‐BV421 (Clone G043H7; Cat#353208), anti‐human PD‐1‐BV421 (Clone EH12.2H7; Cat#367422), anti‐human TIM‐3‐BV605 (Clone F38‐2E2; Cat#345017), anti‐human CD39‐APC (Clone A1; Cat#328209), anti‐human LAG‐3‐PE (Clone C9B7W; Cat#369306), anti‐human TIGIT‐PerCP‐CY5.5 (Clone A15153G; Cat#372718), anti‐human IFN‐γ‐PE (Clone W19227A; Cat#383303), anti‐human IFN‐γ‐APC (Clone 4S.B3; Cat#502512), and anti‐human TNF‐α‐PE (Clone MAB11; Cat#502908) were purchased from BioLegend.

### 4.5 Subcutaneous Tumor Models

Mice were subcutaneously injected into the right axilla on day 0 with 1 × 10^6^ MC38 tumor cells, 7 × 10^5^ B16‐F10 tumor cells, 3.5 × 10^6^ 4T1 tumor cells, or 1.2 × 10^6^ LLC tumor cells. Tumor size was measured every two days using vernier calipers and calculated as (length × width^2^) / 2.

For DADA treatment, mice received DADA (Cat#D50741G, TCI, USA) in their drinking water at a dose of 50 mg kg^−1^ starting from day ‐14, until the experimental endpoints or until day 0. The DADA‐containing drinking water was refreshed twice weekly to ensure a stable drug concentration.

For immune checkpoint blockade (ICB) treatment, mice were intraperitoneally injected with 100 µg of anti‐mouse PD‐1 monoclonal antibody (clone RMP1‐14, Selleckchem, USA) every three days, starting from day 9.

For immune cell depletion experiments, mice were subcutaneously injected with 3 × 10^5^ MC38 tumor cells and received intraperitoneal injections of depleting antibodies starting from day −1 and continuing once weekly thereafter. CD8^+^ T cells were depleted using 100 µg of anti‐CD8α antibody (clone 2.43, Selleckchem, USA). CD4^+^ T cells were depleted using 100 µg of anti‐mouse CD4 antibody (clone GK1.5, Selleckchem, A2101). NK cells were depleted using 100 µg of anti‐asialo GM1 antibody (rabbit polyclonal, FUJIFILM Wako Pure Chemical Corporation, 986–10001).

### 4.6 Metastatic Tumor Model

7 × 10^5^ B16‐F10 cells were injected into the mice via the tail vein on day 0. Mice received DADA (Cat#D50741G, TCI, USA) in their drinking water at a dose of 50 mg kg^−1^ starting from day ‐14, until the experimental endpoints or until day 0. The DADA‐containing drinking water was refreshed twice weekly to ensure a stable drug concentration. Mice were euthanized on day 17, and lung metastatic nodules and lung weights were quantified.

### 4.7 Cell Isolation

For isolation of splenic lymphocytes, the spleens were pressed through sieves, and lymphocytes were then obtained after lysing erythrocytes. For isolation of lymph node lymphocytes, lymph nodes were pressed through sieves, and cell suspensions were collected. For isolation of tumor‐infiltrating lymphocytes, fresh tumor tissues were collected, minced into pieces, and digested in RPMI 1640 medium containing 1 mg mL^−1^ Collagenase IV and 0.2 mg mL^−1^ DNase I at 37°C for 30 min with constant shaking. The digested cell suspension was filtered through a 70 µm cell sieves at 4°C, followed by centrifugation at 300 *g* for 5 min. The supernatant was discarded, and the pellet was resuspended with 3 mL of 40% Percoll. The suspension was then layered onto 2 mL of 70% Percoll and centrifuged at 1260 *g* for 30 min at 25°C to isolate lymphocytes. The lymphocytes were subsequently resuspended in 1 mL of PBS and counted to obtain the total number of recovered TILs per tumor. These cells were subsequently analyzed by flow cytometry to determine the percentage of CD8^+^ T cells among TILs. The absolute number of CD8^+^ T cells per tumor was calculated by multiplying the total TIL count by the CD8^+^ T cell frequency for each individual sample.

For isolation of mouse CD8^+^ T cells, cells were purified from splenic lymphocytes using through magnetic activated cell sorting (MACS) using the Mouse CD8^+^ T Lymphocyte Negative Selection Kit (Cat#130‐104‐075, Miltenyi Biotec, USA).

For isolation of human CD8^+^ T cells, peripheral blood from healthy donors were loaded onto a Ficoll density gradient to isolate peripheral blood mononuclear cells (PBMCs), followed by negative selection using the Human CD8^+^ T Cell Negative Selection Kit (Cat#P8680, Solarbio, Beijing) via MACS. The purity of the sorted cell populations was > 90%.

### 4.8 Flow Cytometry

The isolated cells were incubated with anti‐CD16/32 monoclonal antibody (Cat#560539, BD Bioscience, USA) to block Fc receptors, followed by incubation with fluorescence antibodies against surface molecules and a live/dead viability dye (Cat#423113, BioLegend, USA) for 30 min at 4°C. For intracellular staining, cells were stimulated with phorbol ester (50 ng mL^−1^, TargetMol), ionomycin (1 µg mL^−1^, TargetMol), and monensin (5 µg mL^−1^, TargetMol) for 4 h at 37°C. After surface molecule staining, cells were fixed and permeabilized using a fixation/permeabilization kit (Cat#424401, BioLegend, USA) prior to intracellular molecule staining. All stained samples were analyzed by a CytoFLEX flow cytometer (Beckman Coulter, USA), and data were processed using FlowJo v10.8.1 software.

### 4.9 In Vitro CD8^+^ T Cell Activation and Functional Analysis

For in vitro activation, purified mouse CD8^+^ T cells were stimulated with anti‐CD3 (5 µg/mL, Cat#830301, BioLegend, USA) and anti‐CD28 (2.5 µg mL^−1^, Cat#102103, BioLegend, USA) antibodies, in the presence of DADA (40 µM) or DMSO, for 24 or 48 h, and 1 µM monensin was added during the last 4 h. Cells were then collected and analyzed for IFN‐γ, TNF‐α, and GZMB expression by flow cytometry.

### 4.10 In Vitro Chronic T Cell Stimulation

For induction of exhaustion in mouse CD8^+^ T cells, purified CD8^+^ T cells were stimulated with anti‐mouse CD3 antibody (5 µg mL^−1^), anti‐mouse CD28 antibody (2.5 µg mL^−1^), and recombinant mouse IL‐2 (50 U mL^−1^) in 48‐well plates at a density of 8 × 10^5^ cells per well for 6 days. DADA (40 µM) or DMSO was added into the culture medium starting from day 3. In some experiments, Oligomycin (1 µM; Cat#11342, Sigma–Aldrich, USA), Acetyl‐CoA (2 mM; Cat#A8070, Beijing, China), UK5099 (20 µM; Cat#T4441, TargetMol, USA) were added starting from day 3.

For induction of exhaustion in human CD8^+^ T cells, purified peripheral blood CD8^+^ T cells were stimulated with anti‐mouse CD3 antibody (5 µg mL^−1^), anti‐mouse CD28 antibody (2.5 µg mL^−1^), and recombinant human IL‐2 (200 U mL^−1^) in 24‐well plates at a density of 1 × 10^6^ cells per well for 6 days. DADA (40 µM) or DMSO was added into the culture medium starting from day 3.

For induction of exhaustion in OT‐1 cells, splenic OT‐1 cells were activated initially with OVA peptide (1 µg mL^−1^; Cat#138831‐86‐4, QYAO BIO, China) and recombinant mouse IL‐2 (50 U mL^−1^) in (8 × 10^5^ cells per cell). Following a 2‐day activation period, OT‐1 cells were further stimulated with anti‐mouse CD3 antibody (5 µg mL^−1^), anti‐mouse CD28 antibody (2.5 µg mL^−1^), and recombinant mouse IL‐2 (50 U mL^−1^), in the presence of DADA (40 µM) or DMSO for an additional 4‐day incubation period.

### 4.11 Adoptive T Cell Transfer

MC38‐OVA cells (1 × 10^6^ cells mouse^−1^) were subcutaneously injected into the right axilla of 6‐week‐old C57BL/6J mice. On day 9 post‐injection, tumor‐bearing mice were randomly divided into two groups. CTV (Cat#C34554, Thermo Fisher Scientific, USA) ‐labeled OT‐1 cells (1.5 × 10^6^ cells mouse^−1^), which had undergone an initial activation with OVA peptide for 2 days and subsequent stimulation with anti‐mouse CD3 antibody (5 µg mL^−1^), anti‐mouse CD28 antibody (2.5 µg mL^−1^), and recombinant mouse IL‐2 (50 U mL^−1^) in the presence of DADA (40 µM) or DMSO for 4 days, were intravenously injected into the tumor‐bearing mice. Tumor size was measured every two days using vernier calipers, and was calculated as (length × width^2^)/2. On day 15 post‐tumor inoculation, CTV^+^ CD45.2^+^ T cells were isolated from the tumor and TDLN and analyzed by flow cytometry.

### 4.12 Quantitative Reverse‐Transcription PCR

Total RNA was extracted using TRIzol reagent (Thermo Fisher Scientific) and reverse‐transcribed into cDNA using HiScript reverse transcriptase (Vazyme) according to the manufacturer's instructions. qPCR was performed on a CFX ConnectTM real‐time PCR system (BIO‐RAD, CA, USA) using SYBR Premix Ex Taq (TaKaRa Biotechnology, Dalian, China). Cycling conditions were as follows: 35 cycles of 95°C for 10 s and 60°C for 30 s. All primers were synthesized by General Bio (Anhui) Co. Gene expression levels were normalized to the housekeeping gene *Gapdh*, and relative mRNA expression was calculated using the 2^(‐ΔΔCt) method. The primer pairs used for the genes were as follows:

**Table:** 

Gene	Primer sequence
*Gapdh*‐F	5’‐TCAACAGCAACTCCCACTCTTCCA‐3’
*Gapdh*‐R	5’‐ACCTGTTGCTGTAGCCGTATTCA‐3’
*Tcf7*‐F	5′‐AGCTTTCTCCACTCTACGAACA‐3′
*Tcf7*‐R	5′‐AATCCAGAGAGATCGGGGGTC‐3′
*Ccr7*‐F	5′‐TGTACGAGTCGGTGTGCTTC‐3′
*Ccr7*‐R	5′‐GGTAGGTATCCGTCATGGTCTTG‐3′
*Lef1*‐F	5′‐TGTTTATCCCATCACGGGTGG‐3′
*Lef1*‐R	5′‐CATGGAAGTGTCGCCTGACAG‐3′
*Bach2*‐F	5′‐TCAATGACCAACGGAAGAAGG‐3′
*Bach2*‐R	5′‐GTGCTTGCCAGAAGTATTCACT‐3′
*Cox5a*‐F	5’‐GCCGCTGTCTGTTCCATTC‐3’
*Cox5a*‐R	5’‐GCATCAATGTCTGGCTTGTTGAA‐3’
*Cox7c*‐F	5’‐ATGTTGGGCCAGAGTATCCG‐3’
*Cox7c*‐R	5’‐ACCCAGATCCAAAGTACACGG‐3’
*Pdk1*‐F	5’‐GGCTATGAGAACGCTAGGCG‐3’
*Pdk1*‐R	5’‐TGAACTCTTTAAGAATGCCATG‐3’
*Pdk2*‐F	5’‐GACTCTAAGCCAGTTCACAGATG‐3’
*Pdk2*‐R	5’‐CTTTAAGAATGCCATGCGGGCC‐3’
*Pdk3*‐F	5’‐GTTGAACCGCCCTTCCGTGG‐3’
*Pdk3*‐R	5’‐GGTACATGCAGAGCTTCCTTGA‐3’
*Pdk4*‐F	5’‐GATGCTCGACCGTGGCCCTC‐3’
*Pdk4*‐R	5’‐TTCACCACATGCTCTTCGAACT‐3’

### 4.13 Measurement of Acetyl‐CoA Concentration

Cells were collected, and lysed using an ice‐bath sonicator set at 200 W power. The sonication was performed with 3 s bursts followed by 10 s intervals, repeated 30 times. Cell supernatants were then collected and the concentration of Acetyl‐CoA was measured using an Acetyl‐CoA content assay kit (Cat#BC0980, Solarbio, Beijing, China) according to manufacturer's instructions.

### 4.14 Extracellular Flux Analysis

OCR and ECAR were measured using a Seahorse XF96 extracellular flux analyzer (Agilent Technologies). Briefly, the cells were collected and resuspended in a non‐buffered assay medium with a pH of 7.4. They were then seeded onto the XF96 microplates that had been pre‐treated with poly‐L‐Lysine and incubated in a non‐CO_2_ incubator for 30 min before the assay. For ECAR measurements, the XF glycolysis stress test kit (Cat#103020‐100, Agilent Technologies, USA) was utilized. Following the baseline recordings, 10 mM glucose, 1 µM oligomycin A, and 50 mM 2‐DG were sequentially injected. For OCR measurements, the XF Mito stress test kit (Cat#103015‐100, Agilent Technologies, USA) was employed. Following the baseline recordings, 1 µM oligomycin A, 2 µM FCCP, and a mixture of 0.5 µM rotenone and 0.5 µM antimycin A were sequentially injected.

### 4.15 Mitochondrial Fitness Assay

To analyze the mitochondrial mass and membrane potential, MitoTracker Green and TMRM were used. Prior to cell surface staining, the cells were incubated with 200 nM MitoTracker Green (Cat#M7514, Thermo Fisher) and 50 nM TMRM (Cat#T669, Thermo Fisher) for 15 min at 37°C in a 5% CO_2_ incubator in the dark. Following staining, the cells were washed with PBS and analyzed by flow cytometry.

For measurement of mitochondrial ROS production, cells were collected, adjusted to a density of 1 × 10^6^ cells mL^−1^, and washed three times with PBS. Cells were then incubated with 1 µM MitoSox Red (Cat#M36008, Thermo Fisher) for 30 min at room temperature in the dark. After incubation, cells were washed three times with PBS to remove extracellular probe residue and analyzed by flow cytometry.

### 4.16 Retroviral Vector Construction and Viral Transduction

Self‐inactivating retroviral vector pSIR‐U6‐GFP was generated by modifying pSIR‐dsRed‐Express2 (Addgene #51135), with all BbsI sites were mutated. For cloning sgRNA into the pSIR vector, an annealed sgRNA oligos were directly inserted into BbsI‐digested pSIR‐U6‐GFP by T4 ligation similar to the cloning method utilized by lentiCRISPRv2. sgRNAs were designed by using the online tool (https://portals.broadinstitute.org/gppx/crispick/public). sgRNAs used in this study were as follows: non‐targeting control (NTC) sgRNA, ATGACACTTA CGGTACT CGT; PDK1 sgRNA no.1, ATGGCTATGAGAACGCTAGG; PDK1 sgRNA no.2, ACCATCCCATCTCTATCACA;PDK2 sgRNA no.1, TTAGAGTCCGGTGGTCC TCG; PDK2 sgRNA no.2, GGGACATAGACCATGTGAAT; PDK3 sgRNA no.1, GA ACTAATCCCACGGAAGGG; PDK3 sgRNA no.2, CTTGTTGAACCGCCCTTCCG; PDK4 sgRNA no.1, ATGCTCGACCGTGGCCCTCA; PDK4 sgRNA no.2, TAT CGACCCAAACTGTGATG. Purified splenic Cas9 CD8^+^ T cells were activated with 5 µg mL^−1^ anti‐CD3 and 2.5 µg mL^−1^ anti‐CD28 for 24 h before viral transduction. Retroviral transduction was performed by spin‐infection at 1200 *g* at 37 °C with 10 µg mL^−1^ polybrene (Solarbio) for 1.5 h. pSIR‐U6‐GFP retroviral vector, which was co‐transfected into Plat‐E cells with the helper plasmid pCL‐Eco (Addgene no. 12371) for the production of retrovirus.

### 4.17 Lentivirus Production

293 T cells were cultured to 80%–90% confluence for the production of lentiviral particles carrying chimeric antigen receptor (CAR). These cells were co‐transfected with three plasmids: the packaging plasmid psPAX2, the envelope plasmid pMD2.G, and the CAR plasmid, using jetPRIME reagent (Polyplus, Marguerite Perey, France). 6–8 h post‐transfection, the medium was replenished, and lentiviral supernatants were collected at 48 and 72 h. The collected lentiviral supernatants were filtered through a 0.45 µm polyethersulfone (PES) membrane filter (BS‐PES‐45, Biosharp, China) to remove cellular debris.

### 4.18 Generation of CAR‐T Cells

Activated human T cells were transduced with lentivirus carrying CAR in the presence of 8 µg mL^−1^ polybrene (Solarbio) at multiplicities of infection (MOIs) of 5 and 10. To optimize transduction efficiency, the plates were centrifuged at 800 *g* for 1.5 h at 37°C. The lentivirus‐containing medium was replenished 6–8 h post‐infection to remove the lentivirus, and substituted with medium supplemented with recombinant human IL‐2 (200 U mL^−1^). On day 2 post‐transduction, CAR expression was quantified using GFP as a reporter through flow cytometry, with a transduction efficiency of approximately 50%–60%. All analyses associated with CAR‐T cells were gated from CD3^+^CAR^+^ cells. The DADA (40 µM) was added after CAR‐T cell production. Fresh medium containing DADA was replenished every 2 days during the CAR‐T cell culture period.

### 4.19 Repeated Stimulation of CAR‐T Cells

CAR‐T cells were pretreated with DADA or DMSO for 4 days. Following this treatment, the DADA or DMSO was removed, and the CAR‐T cells were co‐cultured with Raji cells in 24‐well plates at an effector‐to‐target (E:T) ratio of 1:1. Every 48 h, CAR‐T cells were harvested, counted, and re‐stimulated with freshly added Raji cells at the same initial E:T ratio. DADA was absent during all phases of Raji cell stimulation. Following 3 rounds of co‐culture with the Raji cells, the CAR‐T cells were collected for analysis of exhaustion and effector functions using flow cytometry.

### 4.20 In Vivo CAR‐T Cell Therapy

NCG mice were intravenously injected with 5 × 10^5^ Raji‐Luc cells on day 0. On day 7 post‐injection, tumor‐bearing mice were randomly divided into three groups with similar tumor burden. CAR‐T cells (1 × 10^6^ cells mouse^−1^), which had pretreated with DADA (40 µM) or DMSO for 4 days, were intravenously injected into the tumor‐bearing mice. Tumor burden and survival were monitored using bioluminescence imaging (BLI) every 3–4 days until the experimental endpoints.

### 4.21 Single‐Cell RNA‐Sequencing

Single‐cell transcriptome sequencing was conducted by Shanghai Boho Biotechnology Co. Specifically, single‐cell capture and library construction were performed using the 10× Genomics Chromium single‐cell system (10× Genomics, Pleasanton, CA, USA). Sequencing was carried out on an Illumina NovaSeq 6000 platform (Illumina, San Diego, CA, USA) using paired‐end 150 bp (PE150) reads, with an average of ∼50 000 high‐quality reads per cell. Raw sequencing data (fastq format) underwent rigorous quality control using Cell Ranger software (10× Genomics, v7.1.0) and were aligned to the reference genome (mm10/hg38).

### 4.22 Analysis of Single‐Cell RNA‐Sequencing Data

Raw single‐cell RNA‐sequencing reads were aligned to the mm10 mouse reference genome using Cell Ranger. After generating a gene expression matrix via Cell Ranger Count, cells were subjected to quality control (QC) to retain high‐quality cells with unique feature counts (gene numbers) ranging from 1000 to 6000 and a mitochondrial gene expression ratio ≤5%. Data integration was performed based on 2000 highly variable genes (HVGs) using the IntegrateData function of Seurat v4.0.6. The gene expression matrix was then normalized to remove the effects of mitochondrial gene proportion and sequencing depth on expression. After dimensionality reduction by principal component analysis (PCA), cells were clustered using Louvain's algorithm at a resolution of 0.5. Unidentified cell types were annotated using the SingleR R package, which leverages a single‐cell reference expression dataset. By calculating the Spearman correlation between the expression profiles of query cells and annotated cells in the reference dataset, SingleR assigns the reference cell type with the highest correlation to each query cell, minimizing subjective bias and ensuring objectivity in cell type identification. The raw sequencing data have been deposited in the Gene Expression Omnibus (GEO) database under the accession number GSE317913.

### 4.23 RNA‐Sequencing

CD8^+^ T cells isolated from the spleens were stimulated with anti‐mouse CD3 antibody (5 µg mL^−1^), anti‐mouse CD28 antibody (2.5 µg mL^−1^), and recombinant mouse IL‐2 (50 U mL^−1^) at a density of 1 × 10^6^ cells mL^−1^ for 6 days. DADA (40 µM) or DMSO was added into the culture medium starting from day 3. On day 6, the cells were collected, and total RNA was extracted using TRIzol reagent. RNA quality was assessed by NanoDrop2000 and Agilent 2100, confirming concentrations ≥200 ng µL^−1^ and RIN values ≥7.0. A cDNA library was constructed using the Multi‐Stranded mRNA‐Seq kit (DynaPro Bio) via end repair, adaptor ligation, and PCR amplification, followed by PE150 sequencing on an Illumina HiSeq X Ten platform (single‐sample data volume ≥6 Gb). RNA‐Seq data analysis was conducted by Nanjing Kinko Biotechnology Co. The raw sequencing data have been deposited in the Gene Expression Omnibus (GEO) database under the accession number GSE317958.

### 4.24 Statistical Analysis

Statistical significance was determined using Prism 10.0 (GraphPad Software). Data are presented as mean ± SD. For comparisons between two groups, a two‐tailed Student's t‐test was used. In cases involving more than two groups, one‐way ANOVA or two‐way ANOVA was used. Dynamic changes across groups were analyzed using two‐way ANOVA. *P* < 0.05 was considered statistically significant: *p < 0.05, **p < 0.01, ***p < 0.001, ****p < 0.0001. Details regarding sample sizes, the times of repeated experiments, and specific statistical tests are provided in the figure legends.

## Author Contributions

**J.C**., **W.X.F**., and **W.X.W**. conceived the project. **M.B**., **F.L**., and **H.J**. designed and performed experiments, analyzed data, and wrote the manuscript. **L.G**., **M.S**., **K.L**., **P.L**., **R.X**., and **W.M**. performed experiments. **M.Z**. collected human samples. **J.C**., **W.X.F**. and **W.X.W**. wrote and revised the manuscript, and supervised the project.

## Conflicts of Interest

The authors declare no conflicts of interest.

## Supporting information




**Supporting File**: advs74579‐sup‐0001‐SuppMat.docx.

## Data Availability

The data that support the findings of this study are available from the corresponding author upon reasonable request.
