# Supplementary material for: DADA Enhances CD8+ T Cell Stemness to Improve Anti‐Tumor Immunity and Immunotherapy Efficacy
Source: Adv Sci (Weinh). 2026 Feb 27;13(26):e19765. doi: 10.1002/advs.202519765 (PMC13159152; doi:10.1002/advs.202519765)
Supplement: Supplementary file 1 — Supporting File: advs74579‐sup‐0001‐SuppMat.docx. [file ADVS-13-e19765-s001.docx]

**Supporting Information for**

**DADA enhances CD8^+^ T cell stemness to improve anti-tumor immunity and immunotherapy efficacy**

Mingyue Bi^1,2,6^, Fei Li^2,6^, Heng Jiang^3,6^, Lingling Gu^2^, Mengjie Sun^2^, Kunyu Lu^2^, Pianpian Liu^4^, Ruyue Xie^2^, Wenqiang Ma^2^, Meijuan Zheng^1^, Xianwei Wang^5^*, Xuefu Wang^2^*, Jingjing Cong^1,2,7^*

^1^ Department of Clinical Laboratory, First Affiliated Hospital of Anhui Medical University; Hefei, Anhui, China

^2^ School of Pharmacy, Inflammation and Immune Mediated Diseases Laboratory of Anhui Province, Anhui Medical University; Hefei, Anhui, China

^3^ Department of General Surgery, The Second Affiliated Hospital of Anhui Medical University, Hefei, China.

^4^ Department of Clinical Laboratory, The Second Affiliated Hospital of Anhui Medical University, Hefei, China

^5^ State Key Laboratory of Immune Response and Immunotherapy, the Institute of Immunology, School of Basic Medical Sciences, Division of Life Sciences and Medicine, University of Science and Technology of China; Hefei, Anhui China

^6^ These authors contributed equally to this work.

^7^ Lead contact

* Xianwei Wang, Xuefu Wang, and Jingjing Cong

Email: congjingjing@ahmu.edu.cn, wangxuefu@ahmu.edu.cn, or wangxianwei@ustc.edu.cn

**Figure S1. DADA potentiates CD8^+^ T cell effector functions in the TDLN.**

(A) WT mice were fed with DADA-containing or normal drinking water, and body weight was monitored; n = 6.

(B) Representative H&E-stained sections of heart, liver, spleen, lung, and kidney tissues from mice in the Ctrl and DADA-treated groups.

(C) Gating strategy for the flow cytometry analyses of *in vivo* isolated CD8^+^ T cells.

(D) Quantification of IFN-γ^+^ and TNF-α^+^ cells among CD8^+^ T cells from TDLN of B16-F10 (s.c.) tumor-bearing mice; n = 6. The experiment was repeated twice.

(E) Quantification of IFN-γ^+^ and TNF-α^+^ cells among CD8^+^ T cells from TDLN of MC38 (s.c.) tumor-bearing mice; n = 6. The experiment was three times.

(F) Quantification of IFN-γ^+^ and GZMB^+^ cells among CD8^+^ T cells from TDLN of B16-F10 (i.v.) tumor-bearing mice; n = 7. The experiment was repeated twice.

(G) Schematic experimental procedure for (H)-(K): WT mice were fed with DADA-containing or normal water for 14 days and were injected (i.p.) with 100 μg of anti-CD4 or ASGM1 antibody weekly. The mice were injected (s.c.) with MC38 cells on day 0.

(H-K) Tumor growth curves (H), images of tumors (I), Tumor weights (J), and representative flow cytometry plots of CD4^+^ T cells and NK cells (K) at 17 days after MC38 inoculation; n = 6. Data are presented as mean ± SD and are analyzed by two-way ANOVA (A and H), and one-way ANOVA (D-F and J); *p < 0.05, **p < 0.01, ***p < 0.001, ****p < 0.0001. ns, not significant.

**Figure S2. DADA prevents terminal exhaustion of CD8^+^ T cells in the TDLN.**

(A) Biaxial tSNE plots showing clustering of tumor-infiltrating CD45⁺ cells in the Ctrl and DADA-treated groups.
(B) Biaxial tSNE plots showing secondary clustering of CD8^+^ T cells in the Ctrl and DADA-treated groups.

(C) Quantification of PD-1⁺TIM-3⁺, TCF1⁻TIM-3⁺, and Ki67⁺ cells among CD8^+^ T cells from TDLN of MC38 (s.c.) tumor-bearing mice; n = 6. The experiment was repeated twice.

(D) Schematic experimental procedure for (E) and (F): WT mice were fed with DADA-containing or normal water from day -14 until the experimental endpoints or until day 0, and were injected (s.c.) with B16-F10 cells on day 0.

(E and F) Quantification of PD-1⁺TIM-3⁺, TCF1⁻TIM-3⁺, TCF1⁺TIM-3⁻, and Ki67⁺ cells among CD8^+^ T cells from tumor (E) and TDLN (F); n = 6. The experiment was repeated twice.

(G) Schematic experimental procedure for (H) and (I): WT mice were fed with DADA-containing or normal water from day -14 until the experimental endpoints or until day 0, and were injected (i.v.) with B16-F10 cells on day 0.

(H and I) Quantification of PD-1⁺TIM-3⁺, TCF1⁻TIM-3⁺, TCF1⁺TIM-3⁻, TOX⁺, and Ki67⁺ cells among CD8^+^ T cells from tumor (H) and TDLN (I); n = 7. The experiment was repeated twice. Data are presented as mean ± SD and are analyzed by one-way ANOVA (C, E, F, H and I); *p < 0.05, **p < 0.01, ***p < 0.001, ****p < 0.0001. ns, not significant.

**Figure S3. DADA does not directly promote CD8^+^ T cell activation.**

(A) Purified splenic CD8⁺ T cells were stimulated with anti-CD3/CD28 mAbs and IL-2 from for 6 days, with the addition of DADA at the indicated concentrations staring from day 3. Quantification of PD-1⁺TIM-3⁺, TCF1⁻TIM-3⁺, TCF1⁺TIM-3⁻, TNF-α^+^ and IFN-γ^+^ and GZMB^+^ cells among CD8^+^ T cells; n = 3. The experiment was repeated twice.
(B) Schematic experimental procedure for the generation of mouse exhausted CD8⁺ T cells for (C): purified splenic CD8⁺ T cells were stimulated with anti-CD3/CD28 mAbs and IL-2 from for 6 days, with DADA (40 μM) or DMSO added throughout the activation and exhaustion phases.
(C) Quantification of PD-1⁺TIM-3⁺, Ly108, TCF1, KI67 expression, TNF-α^+^, IFN-γ^+^ GZMB^+^, and perforin^+^ cells among CD8^+^ T cells; n = 3.
    (D and E) Purified splenic CD8⁺ T cells were stimulated with anti-CD3/CD28 mAbs and IL-2, in the presence of DADA at the indicated concentrations for 24 (B) or 48 (C) hours. Quantification of TNF-α⁺, IFN-γ⁺, and GZMB⁺ cells among CD8⁺ T cells; n = 3. The experiment was repeated twice.

(F) Schematic experimental procedure for the generation of human exhausted CD8⁺ T cells for (E)-(H): purified peripheral blood CD8⁺ T cells were stimulated with anti-CD3/CD28 mAbs and IL-2 from for 6 days, with the addition of 40 μM DADA starting from day 3.

(G-J) Quantification of CD62L⁺ (E), CCR7 expression (F), TIM-3⁺, TIGIT⁺ (G), IFN-γ^+^ and GZMB^+^ (H) cells among CD8^+^ T cells; n = 3. The experiment was repeated twice.

(K) mRNA levels of the indicated molecules in mouse *Pdk1* knockout CD8⁺ T cells; n = 3.

(L) Mouse *Pdk1* overexpression CD8⁺ T cells induced to exhaustion subjected to DADA treatment were collected. Quantification of TCF1⁻TIM-3⁺, TCF1⁺TIM-3⁻, Ly108 expression, TNF-α^+^, IFN-γ^+^ and GZMB^+^ cells among CD8^+^ T cells; n = 3.

(M) mRNA levels of the indicated molecules in mouse *Pdk1* overexpression CD8^+^ T cells; n = 3.

(N) mRNA levels of the indicated molecules in mouse *Pdk2,* *Pdk3*, or *Pdk4* knockout CD8⁺ T cells; n = 3.

(O) Mouse *Pdk1*, *Pdk2*, *Pdk3*, or *Pdk4* knockout CD8⁺ T cells induced to exhaustion subjected to DADA treatment were collected. Quantification of PD-1⁺TIM-3⁺, Ly108, expression, TNF-α^+^, IFN-γ^+^, and GZMB^+^ cells among CD8^+^ T cells; n = 3. Data are presented as mean ± SD and are analyzed by one-way ANOVA (A, D, E, L and O), unpaired t test (C, K, M and N) and paired t test (G-J); *p < 0.05, **p < 0.01, ***p < 0.001, ****p < 0.0001. ns, not significant.

**Figure S4. DADA improves CD8^+^ T cell mitochondiral fitness.**

(A) Immunoblotting of H3K27ac of DADA- or DMSO-treated mouse exhausted CD8+ T cells. Total histone H3 was used as a loading control.
(B) Quantification of TMRM, MitoTracker, and MitoSox in mouse exhausted CD8⁺ T cells subjected to DADA and Oligomycin combined treatment or single treatment; n = 3. The experiment was repeated three times.
(C) Quantification of TMRM, MitoTracker, and MitoSox in mouse exhausted CD8⁺ T cells subjected to DADA and Acetyl-CoA combined treatment or single treatment; n = 3. The experiment was repeated three times.

(D) Quantification of TMRM, MitoTracker, and MitoSox in mouse exhausted CD8⁺ T cells subjected to DADA and UK5099 combined treatment or single treatment; n = 3. The experiment was repeated three times.Data are presented as mean ± SD and are analyzed by two-way ANOVA (B-D); *p < 0.05, **p < 0.01, ***p < 0.001, ****p < 0.0001. ns, not significant.

**Figure S5. The combination of DADA with ACT or ICB therapy reshapes the differention and effector functions of CD8^+^ T cell in the TDLN.**

(A) Quantification of Ki67⁺, PD-1⁺TIM-3⁺, TCF1⁺TIM-3⁻, IFN-γ⁺, TNF-α⁺, and GZMB^+^ cells among CD8⁺ T cells from TDLN of MC38-OVA (s.c.) tumor-bearing mice receiving ACT therapy; n = 6.
(B) Schematic experimental procedure for (C–F): WT mice were fed with DADA-containing or normal water starting from day -14 until day 0, and were injected (s.c.) with 4T1 cells on day 0. Anti-PD-1 mAb was injected (i.p.) as indicated.

(C and D) Tumor growth curves (C), and Tumor weights (D) at 13 days after 4T1 inoculation; n = 6.

(E and F) Quantification of CD8^+^ T (E), Ki67⁺ CD8⁺ T, PD-1⁺TIM-3⁺ CD8⁺ T, TCF1⁻TIM-3⁺ CD8⁺ T, TCF1⁺TIM-3⁻ CD8⁺ T, IFN-γ⁺ CD8⁺ T, TNF-α⁺ CD8⁺ T and GZMB^+^CD8^+^ T (F) cells from tumor; n = 6.

(G) Schematic experimental procedure for (H–L): WT mice were fed with DADA-containing or normal water starting from day -14 until day 0, and were injected (s.c.) with LLC cells on day 0. Anti-PD-1 mAb was injected (i.p.) as indicated.

(H and I) Tumor growth curves (H), and Tumor weights (I) at 21 days after LLC inoculation; n = 6.

(J and K) Quantification of CD8^+^ T (L), Ki67⁺ CD8⁺ T, PD-1⁺TIM-3⁺ CD8⁺ T, TCF1⁻TIM-3⁺ CD8⁺ T, TCF1⁺TIM-3⁻ CD8⁺ T, IFN-γ⁺ CD8⁺ T, TNF-α⁺ CD8⁺ T and GZMB^+^CD8^+^ T (M) cells from tumor; n = 6.

(L) Quantification of Ki67⁺, PD-1⁺TIM-3⁺, TCF1⁻TIM-3⁺, TCF1⁺TIM-3⁻, IFN-γ⁺, and TNF-α⁺ cells among CD8⁺ T cells from TDLN of MC38 (s.c.) tumor-bearing mice; n = 7. The experiment was repeated three times.

(M) Quantification of Ki67⁺, IFN-γ⁺, and TNF-α⁺ cells among CD8⁺ T cells from TDLN of B16-F10 (s.c.) tumor-bearing mice; n = 7. The experiment was repeated three times.Data are presented as mean ± SD and are analyzed by unpaired t test (A), two-way ANOVA (C and H); one-way ANOVA (D-F, and I-M); *p < 0.05, **p < 0.01, ***p < 0.001, ****p < 0.0001. ns, not significant.

**Figure S6. DADA enhances CD8^+^ CAR-T cell stemness.**

(A) Schematic of the lentiviral vector pCDH-CMV-CAR-EF1α-CopGFP.

(B) Flow cytometry of CAR and GFP expression in CD3⁺ T cells. CAR was detected by anti–218 linker antibody (Linker-PE) and GFP.

(C) Gating strategy for flow cytometry analyses of CAR-T cells in vitro and in vivo.
(D and E) Quantification of CD62L^+^, cells among CD8⁺ and CD4⁺ CAR-T cells from the same batch of CAR-T products; n = 4.

(F and G) Quantification of PD-1^+^, LAG-3^+^, TIM-3^+^, CD39^+^, IFN-γ^+^, TNF-α^+^ , and GZMB^+^, cells among CD8⁺ and CD4⁺ CAR-T cells from the same batch of CAR-T products; n=5.

(H) Quantification of CD62L^+^ CAR-T cells among generated from purified human CD8^+^ T cells; n=5.

(I) Quantification of PD-1^+^, TIM-3^+^, LAG-3^+^, CD39^+^, IFN-γ^+^, TNF-α^+^ , GZMB^+^, and perforin^+^ CAR-T cells among generated from purified human CD8^+^ T cells; n = 5.

(J) Representative H&E-stained sections of heart, liver, spleen, lung, and kidney tissues from mice in the DADA- or DMSO-pretreated CAR-T cells treated groups.

Data are presented as mean ± SD and analyzed by unpaired t test (D–I). *p < 0.05, **p < 0.01, ***p < 0.001, ****p < 0.0001; ns, not significant.
